# Supplementary material for: Unaltered hepatic wound healing response in male rats with ancestral liver injury
Source: Nat Commun. 2023 Oct 10;14:6353. doi: 10.1038/s41467-023-41998-w (PMC10564731; doi:10.1038/s41467-023-41998-w)
Supplement: Supplementary file 1 — Supplementary Information [file 41467_2023_41998_MOESM1_ESM.pdf]

Supplementary Information for

Unaltered hepatic wound-healing response in male rats with ancestral liver injury

Beil, Perner, Pfaller *et al*

This PDF file includes:

Supplementary Figures 1-8

Supplementary Methods

Original study protocol including Pedigree tracing: This document provides original internal study protocol content, with detailed study information

a

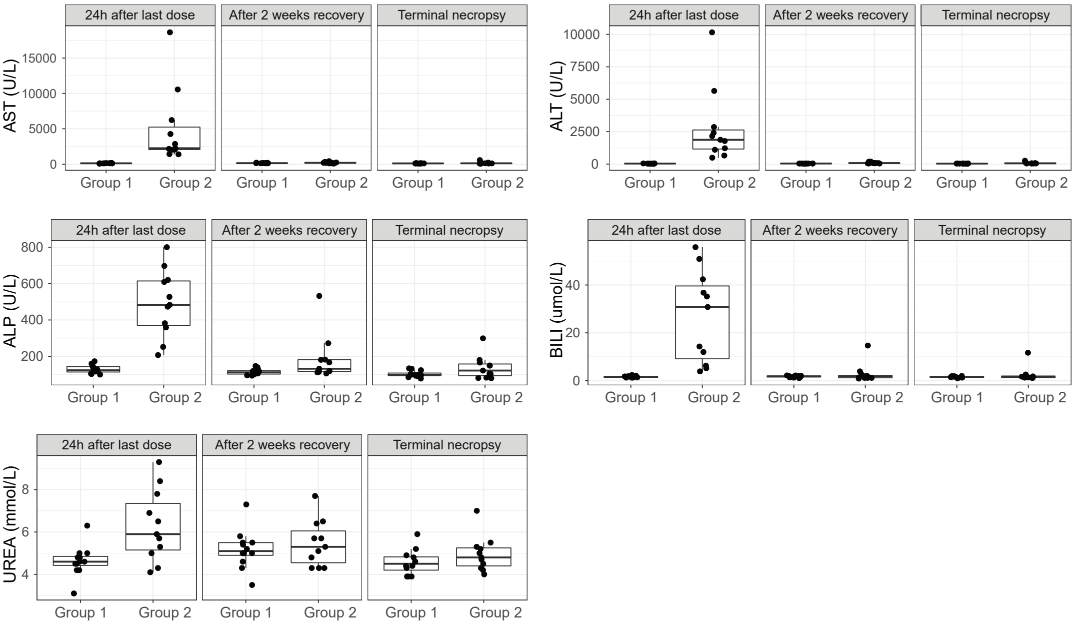

b

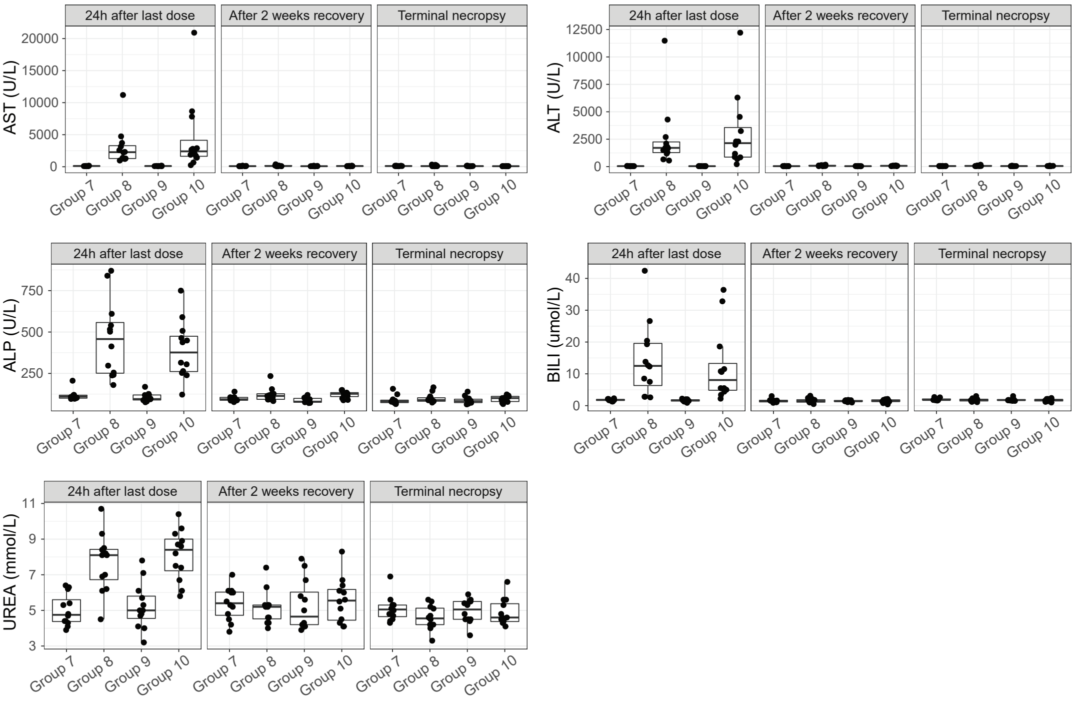

Supplementary Fig. 1 - Clinical biochemistry evaluation of liver injury biomarkers in CCl_4-treated F0 and F1 animals

a, Serum biochemistry evaluation of liver injury biomarkers AST, ALT, total bilirubin (BILI), ALP and urea, in F0 animals and at all three sampling timepoints: 24 hours after last dose, after two weeks recovery and at terminal necropsy (n=12 for group 1, n=11 for group 2). **b,** Serum biochemistry evaluation of liver injury biomarkers AST, ALT, BILI, ALP and urea in F1 animals and at all three sampling timepoints as above (n=12 per group). For all box plots, the median (central line) and the lower and upper quartiles (box limits) are displayed. Whiskers extend to the maximal and minimal value or, if exceeded, to max. the 1.5 × inter-quartile range. Black points represent individual animal values of one sample per animal. Source data are provided as a **Source Data** file.

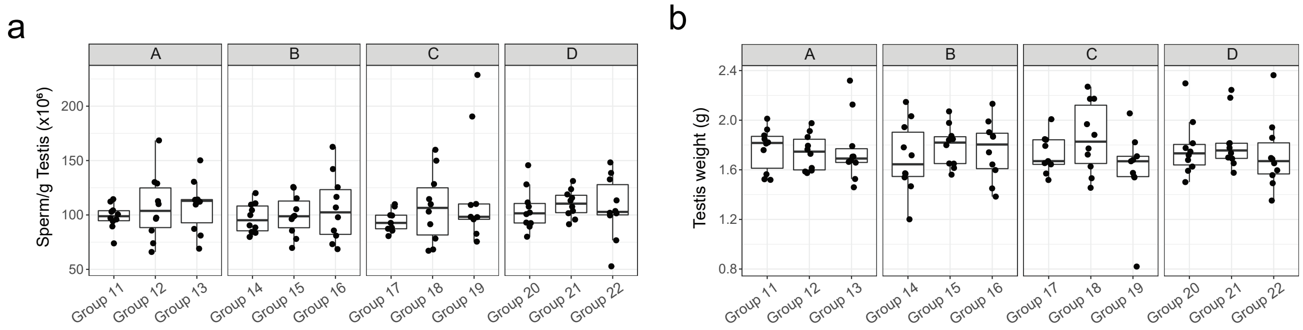

Supplementary Fig. 2 - Evaluation of testicular sperm counts in F2 upon CCl_4 treatment
a, Sperm count per gram testis of F2 animals grouped by cohort. No significant change in sperm count per gram testis across dose groups and within each cohort by Kruskal-Wallis test, p.adj > 0.05. **b,** Weight of left testis in grams for all F2 animals. For all box plots, the median (central line) and the lower and upper quartiles (box limits) are displayed. Whiskers extend to the maximal and minimal value or, if exceeded, to max. the 1.5 × inter-quartile range. Black points represent individual animal values of one sample per animal. Source data are provided as a **Source Data** file.

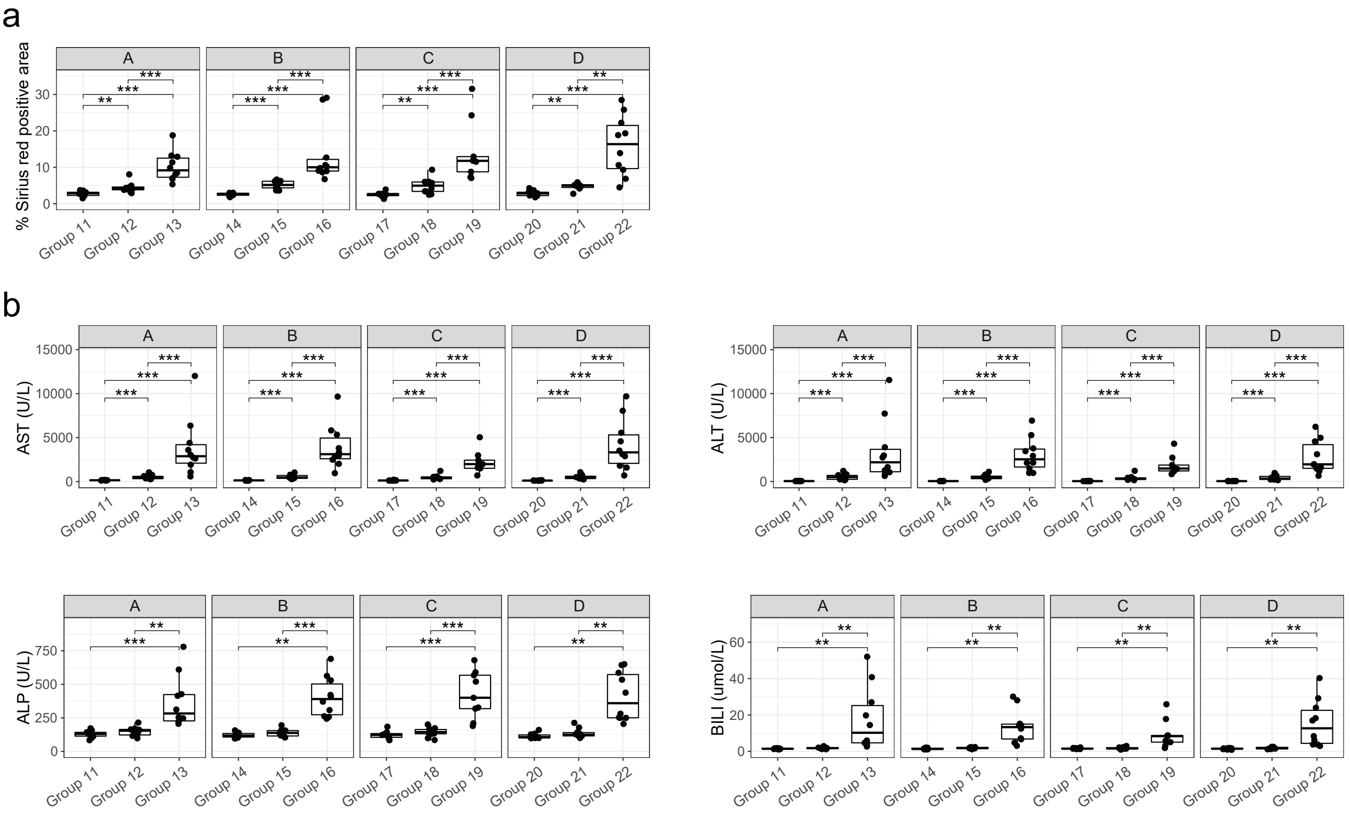

Supplementary Fig. 3 - The presence of liver injury in male ancestors does not reduce liver fibrogenesis in F2 generation male offspring (Sirius Red and serum biochemistry)

a, Sirius Red staining shows similar dose-dependent elevation in Sirius Red positive area for all four cohorts. **b,** Serum biochemistry evaluation of liver injury biomarkers AST, ALT, ALP and total bilirubin (BILI) in F2 samples from all 4 cohorts. Dose-dependent increase in all liver injury markers was observed, in the absence of detectable CCl_4 exposure ancestral effects. For all box plots, the median (central line) and the lower and upper quartiles (box limits) are displayed. Whiskers extend to the maximal and minimal value or, if exceeded, to max. the 1.5 × inter-quartile range. Pairwise two-sided Wilcoxon rank sum test was used for the individual p values indicated as ** p.adj <0.01, *** p.adj <0.001. Black points represent individual animal values of one sample per animal. Exact p values and Source data are provided as a **Source Data** file.

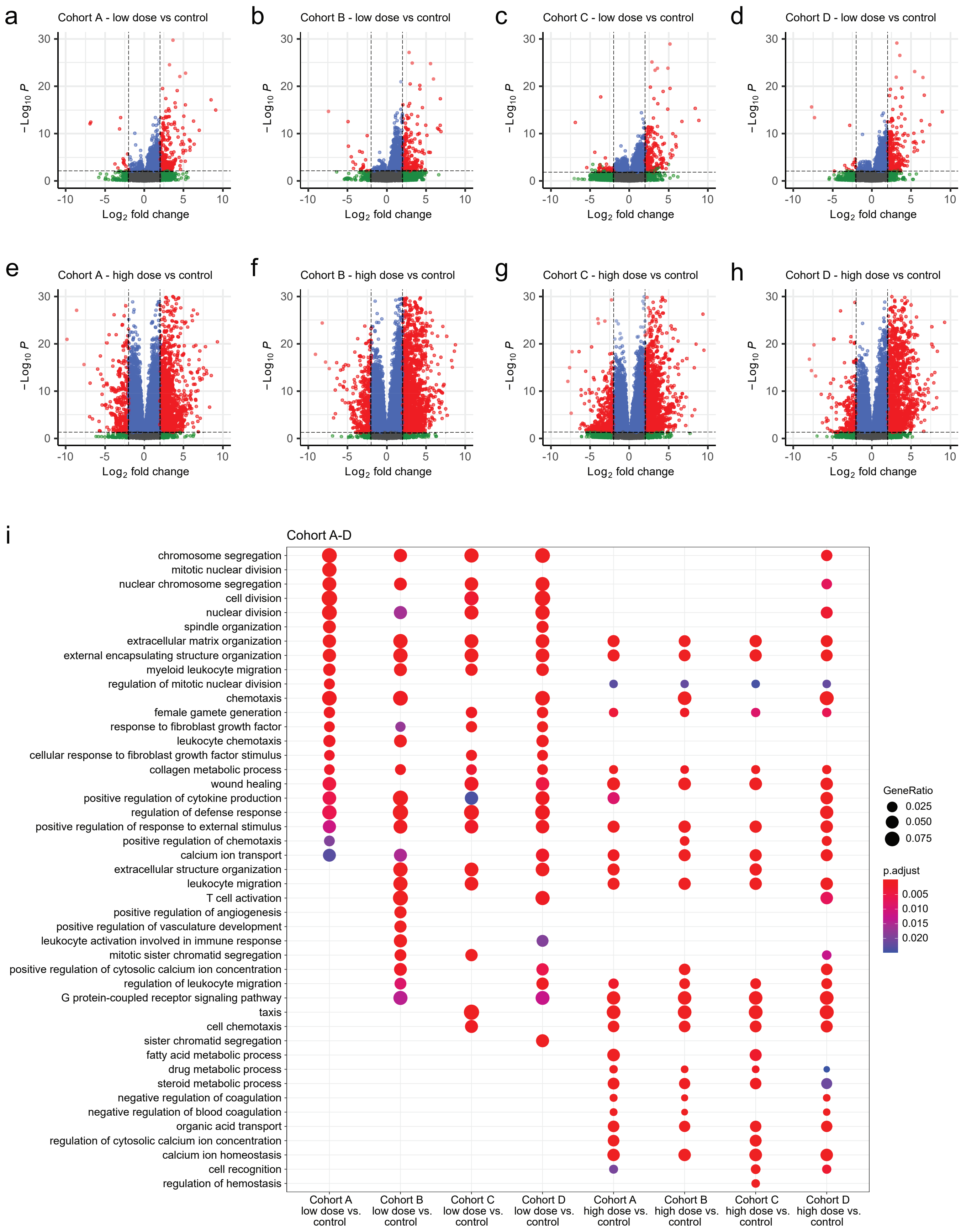
a
Cohort A - low dose vs control
b
Cohort B - low dose vs control
c
Cohort C - low dose vs control
d
Cohort D - low dose vs control
e
Cohort A - high dose vs control
f
Cohort B - high dose vs control
g
Cohort C - high dose vs control
h
Cohort D - high dose vs control
$-\log_{10} P$
$\log_2$ fold change
i
Cohort A-D
chromosome segregation
mitotic nuclear division
nuclear chromosome segregation
cell division
nuclear division
spindle organization
extracellular matrix organization
external encapsulating structure organization
myeloid leukocyte migration
regulation of mitotic nuclear division
chemotaxis
female gamete generation
response to fibroblast growth factor
leukocyte chemotaxis
cellular response to fibroblast growth factor stimulus
collagen metabolic process
wound healing
positive regulation of cytokine production
regulation of defense response
positive regulation of response to external stimulus
positive regulation of chemotaxis
calcium ion transport
extracellular structure organization
leukocyte migration
T cell activation
positive regulation of angiogenesis
positive regulation of vasculature development
leukocyte activation involved in immune response
mitotic sister chromatid segregation
positive regulation of cytosolic calcium ion concentration
regulation of leukocyte migration
G protein-coupled receptor signaling pathway
taxis
cell chemotaxis
sister chromatid segregation
fatty acid metabolic process
drug metabolic process
steroid metabolic process
negative regulation of coagulation
negative regulation of blood coagulation
organic acid transport
regulation of cytosolic calcium ion concentration
calcium ion homeostasis
cell recognition
regulation of hemostasis
Cohort A low dose vs. control
Cohort B low dose vs. control
Cohort C low dose vs. control
Cohort D low dose vs. control
Cohort A high dose vs. control
Cohort B high dose vs. control
Cohort C high dose vs. control
Cohort D high dose vs. control
GeneRatio
0.025
0.050
0.075
p.adjust
0.005
0.010
0.015
0.020

Supplementary Fig. 4 - RNAseq based evaluation of treatment effects on global liver gene expression in F2 animals and across all cohorts

a-h, Volcano plots illustrating differential expression analysis statistics based on DESeq2 analysis (detailed in **Methods**) for low-dose (top row) and high-dose (bottom row) treatment vs control in cohort A-D. For each gene, a point indicates the estimated log2 fold-change (treatment over control group; x-axis) and the associated negative log10 p value (y-axis). Genes are colored based on categorization upon reaching the cutoffs adjusted p value < 0.1 and/or absolute log2 fold-change (Log2 FC) > 1 **i,** Comparison of one-sided GO-term over-representation test results (see **Methods**) between the two dose-contrasts. The dots size (GeneRatio) illustrates the ratio between the number of differentially expressed genes (contrast indicated on x-axis) and the number of GO term-annotated genes. The color scale indicates the FDR (p.adjust) resulting from the over-representation test. Statistics for all dose groups are available in **Supplementary Data 2** and **Source Data**.

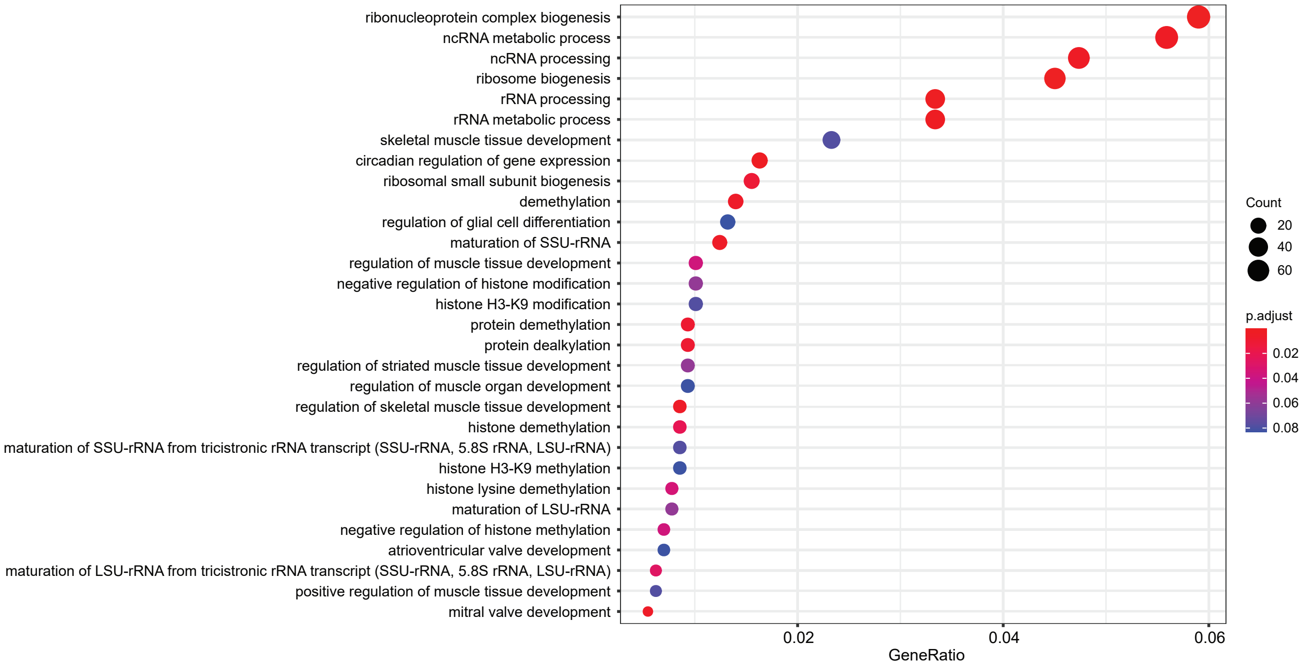

Supplementary Fig. 5 - GO term analysis of the 1523 genes under F0 liver injury influence
Illustration of the one-sided GO term over-representation test results (see **Methods**) based on the 1523 genes signature correlating with F0 treatment history. The x-axis indicates the fraction of GO term-annotated genes that overlap with the gene set. For each over-represented GO term, the dots size (Count) indicates the number of genes from the gene set that overlap with the GO term-annotated genes. The color scale indicates the FDR (p.adjust) resulting from the over-representation test. Statistics for all dose groups are available in **Supplementary Data 2** and **Source Data**.

a

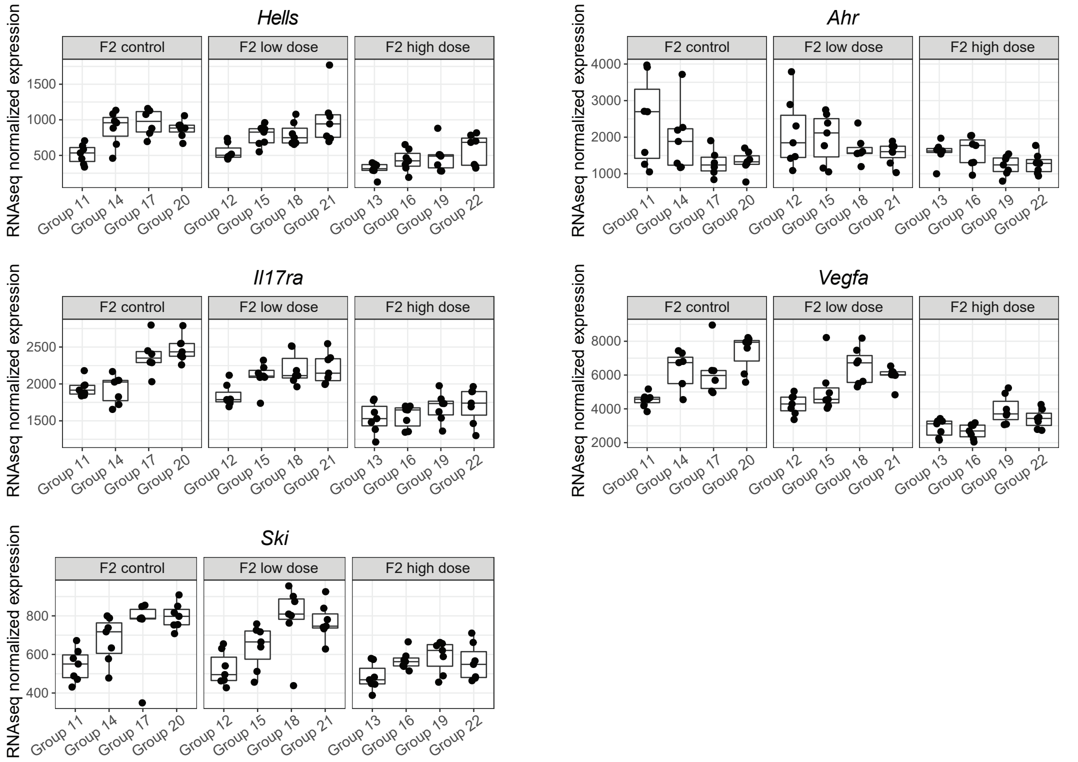

b

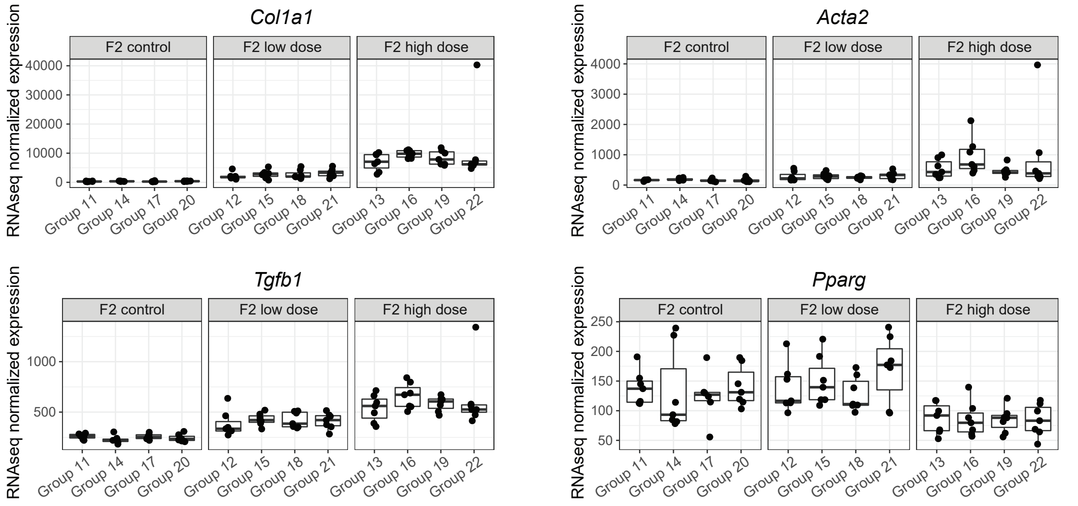

Supplementary Fig. 6 - RNAseq evaluation of exemplary genes under ancestral liver injury versus known fibrosis markers

a, Gene expression measured by RNAseq of exemplary genes with ancestral liver injury signature: lymphoid specific helicase: Hells (LOC100911660), aryl hydrocarbon receptor: Ahr, interleukin 17 receptor A: Il17ra, vascular endothelial growth factor A: Vegfa, and proto-oncogene: Ski (n=7, group 17 n=6). **b,** Gene expression measured by RNAseq of liver fibrosis associated genes: collagen I: Col1a1, alpha smooth muscle actin: Acta2, transforming growth factor β1: TGFb1, and factor peroxisome proliferator-activated receptor γ: Pparg (n=7, group 17 n=6). Visualization of gene expression (y-axis, size-factor normalized counts) for each sample in the F2 generation (black points) by treatment group (x-axis, each box indicated median gene expression, first and third quartiles). For all box plots, the median (central line) and the lower and upper quartiles (box limits) are displayed. Whiskers extend to the maximal and minimal value or, if exceeded, to max. the 1.5 × inter-quartile range. Black points represent individual animal values of one sample per animal. Source data are provided as a **Source Data** file.

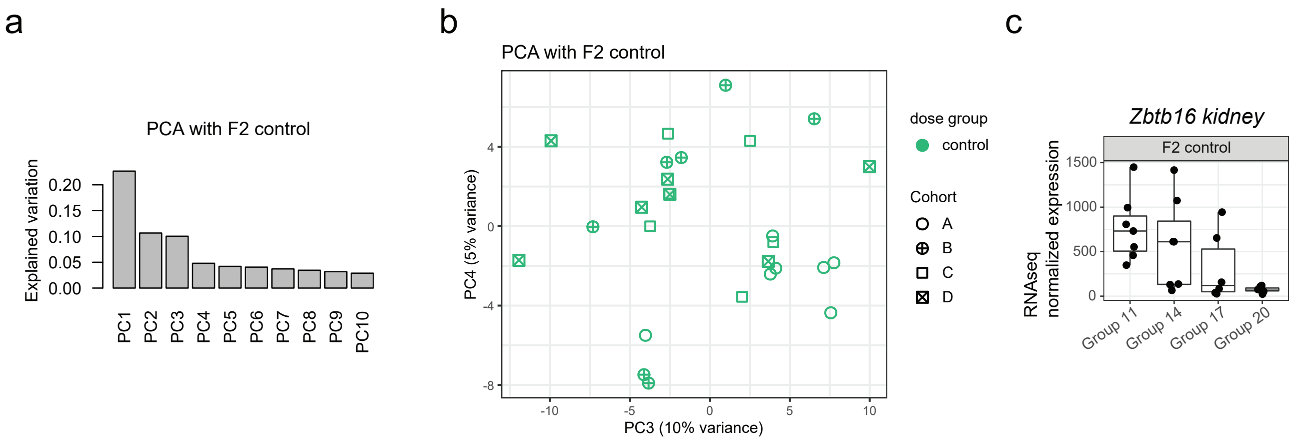

Supplementary Fig. 7 - Cross-tissue (Kidney) RNAseq evaluation of transgenerational CCl_4-mediated liver injury effects

a, PCA analysis giving the variance observed by PC1 to PC10. PC1 and PC2 were excluded as capturing technical variability in kidney tissue processing **b,** PCA plot showing PC3 vs PC4 with a trend of separating of cohort A (group 11, blank circles) samples of naïve origin **c,** Exemplary gene *Zbtb16* under potential F0 influence, showing ancestral exposure related decrease in baseline expression in kidney vehicle treated samples (group 11, 14, 20 n=7, group 17 n=6). Visualization of RNAseq gene expression (y-axis, size-factor normalized counts) for each sample (black points, one sample per animal evaluated) by treatment group across cohorts A-D (x-axis, in each box plot the median (central line) and the lower and upper quartiles (box limits) are displayed. Whiskers extend to the maximal and minimal value or, if exceeded, to max. the 1.5 × inter-quartile range. Black points represent individual animal values of one sample per animal. Source data are provided as a **Source Data** file.). *Zbtb16* was equally found differentially expressed in liver (**Fig 5** and **Fig 6**), potentially highlighting common germline transmitted effects affecting various tissues.

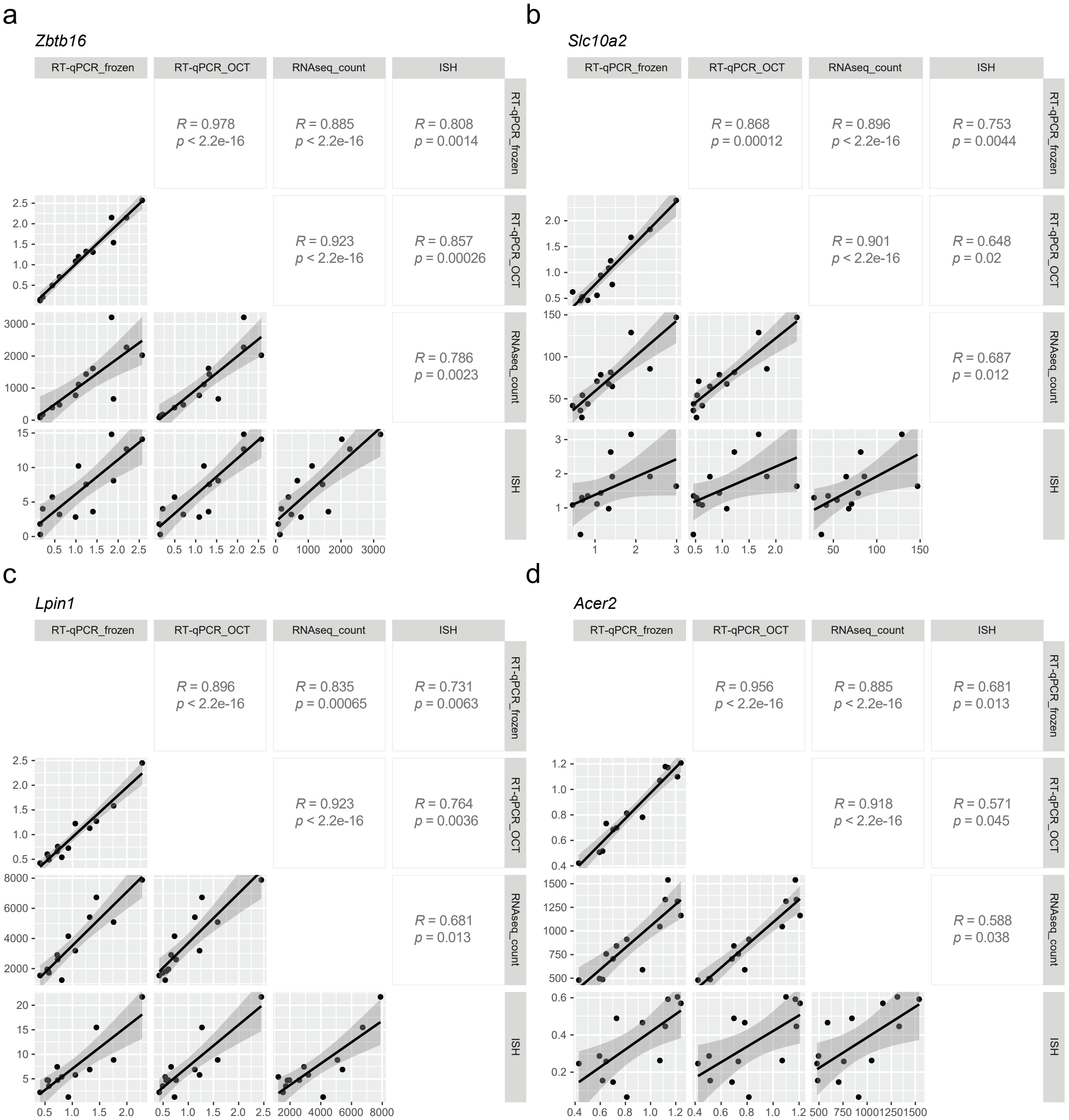

Supplementary Fig. 8 - Cross-readout correlation analyses of a subset of genes influenced by ancestral liver injury in F2 male offspring

a-d, Correlation plots for RNAseq, RT-qPCR from frozen samples, RT-qPCR from OCT samples and ISH data for Zbtb16 (**a**), Slc10a2 (**b**), Lpin1 (**c**), Acer2 (**d**). (group 11 n=7, group 17 n=6). For each animal (points) values on x- and y-axis show measurements from methods indicated in the axis labels. The black line visualizes the linear regression line and the shaded areas the 95% confidence interval regions. Rank based measure of association is estimated using Spearman statistics as indicated by the correlation coefficient (R) and significance level (p).

Supplementary Methods

This document provides original study protocol content, with detailed study information: test article, experimental animals, animal husbandry, treatment, mating, culling. Details on various study schedules are provided for each generation. The sampling and endpoints schemes are provided. The full study pedigree tracing is illustrated and detailed as Appendix 1. The F2 group randomized dosing and necropsy scheme is provided as Appendix 2. This document is complementary to Methods.

A three times weekly carbon tetrachloride (CCl_4) oral (gavage) multi-generational investigational pharmacology and toxicology study in Sprague Dawley rats

Original Study Protocol

The study sponsor is Novartis Pharmaceuticals Corporation, East Hanover, New Jersey 07936. The study is being conducted within the East Hanover facility in accordance with the Novartis Animal Care and Use Committee (NACUC) generic protocol TX 4039.

1 Material and methods

Table 1-1 Control article/vehicle

Control article/vehicle:	Sigma Lot no.
Olive oil	BCBV9066

Table 1-2 Test article

Active ingredient:	Sigma Lot no.
Carbon tetrachloride (CCl_4)	SHBK7371

Table 1-3 Dosage form

Administration form:	Storage conditions:
CCl_4 in olive oil	Room temperature, protect from light
Dose formulations will be prepared approximately weekly.	

1.1 Test system

1.1.1 Experimental animals

Table 1-4 Animal information

Species/ strain	Supplier	No. Ordered	No. assigned to dosing phase	Approximate Age/body weight range (day 1):
Sprague Dawley rat	Charles River Laboratories Raleigh, NC	**F0 phase**:<br>39 males<br>27 females<br>**F1 phase**: 52 females	**F0 Phase**:<br>36 males<br>24 females<br>**F1 phase**: 48 females	10 weeks<br>M - 200 to 350g<br>F - 150 to 300g
Note: Body weights outside the specified ranges may be accepted by the principal Investigator and documented in the data.				

1.1.2 Animal quarters/husbandry/animal identification

Animal caging/animal identification:	Solid bottom caging. 2 or 3 per cage of the same sex except during the mating and lactational periods; 1 male and 1 female co-housed during mating periods; females individually housed with their litters during lactational periods. During gestation, females will be housed 2-3 per cage. On or before Day 20 gestation, animals will be single housed in preparation for parturition. Microchip implants will be used to identify animals on or before Day 21 post partum days of age and older, unless noted otherwise. Prior to 21 days of age, pups will be marked with indelible marker within each litter as needed.

Environmental room conditions (temperature, humidity, lighting), diet, water and enrichment will be performed per facility practices for the test species.

1.2 Study design, animal allocation and activity schedule

1.2.1 Animal allocation

For the F0 generation, animals deemed acceptable for use on study based on the pretest examinations will be randomized to groups via a computer program based on body weight.

For F1 generation selection, males will be randomly selected from available F0 litters on day 21 post partum at the time of weaning. At least two male pups/F0 litter will be selected to comprise the F1 generation. If at least 2 male pups/F0 litter are not available and/or there are not enough F0 litters to select 12 males/group for the F1 generation, additional pups from available litters will be selected (no more than 3 male pups from any one litter). Pup selections will be documented. No more than 1 pup from each F0 litter will be assigned to any F1 dose group, if possible. If insufficient numbers of pups are available, a maximum of 2 pups from a F0 litter may be assigned to an F1 dose group and will be documented in the raw data.

For F2 generation selection, males will be randomly selected from available F1 litters. Three pups/F1 litter will be selected to comprise the F2 generation. If 3 pups/F1 litter are not available and/or there are not enough F1 litters to select 20 pups/group for the F2 generation, additional pups from available litters will be selected (no more than 4 pups from any one litter) and documented. No more than 1 pup from each F1 litter will be assigned to a any F2 dose group, if possible. If insufficient numbers of pups are available, a maximum of 2 pups from a F1 litter may be assigned to an F2 dose group and will be documented in the raw data.

All female animals used in the study were naïve and not derived from F0 or F1 litters.

Animal replacement (if needed), will be documented by the Principal Investigator in the raw data.

The full study pedigree tracing is illustrated and detailed in Appendix 1.

1.2.2 Study design

Duration of CCl_4 administration

Doses will be administered using glass syringes.

F0 males:	Main study (groups 1 and 2): Three times weekly via oral gavage for 6 weeks beginning at 10 weeks of age
F0 females:	Not dosed, naive
F1 males:	Three times weekly via oral gavage for 6 weeks beginning at 9-11 weeks of age
F1 females:	Not dosed, naive
F2 males:	Three times weekly via oral gavage for 6 weeks beginning at 9-11 weeks of age
F2 females:	Not dosed , naive

Mating

Animals in each group will be cohoused 1 female:1 male and mating will continue for up to two weeks. Females will be checked daily for confirmation of mating. Mating will be considered to have occurred when sperm is observed in the vaginal washing and/or a vaginal plug is observed (designated as gestation day 0). Once mating has occurred, females will be returned to their home cages. The mating period may be extended if necessary. The decision to extend the mating period will be made and documented by the principal investigator.

Sibling F1 matings will be avoided and mating pairs will be documented.

At the end of the mating period, sperm-negative females (defined as females for which sperm in the vaginal washing and/or a vaginal plug was not observed) will be returned to their home cages.

Culling

On day 4 post partum (litters may also be culled in the afternoon of day 3 post partum or the morning of day 5 post partum if day 4 post partum is on a weekend) , all F0 and F1 litters will be randomly culled to 8 pups/litter for the F1 and F2 generations, respectively. Male pups will be preferentially retained. If 8 males/litter are not available, female pups will be retained to create a litter of 8 pups. If there are less than 8 pups in a litter, the litter will not be culled. Pups will be individually identified on or before Day 21 post partum with a microchip.

Terminal Euthanasia

All adult animals and pups Day 21 post partum and older will be euthanized via CO_2 or isoflurane anesthesia followed by exsanguination. Any live fetuses resulting from early euthanasia of gestating females will be euthanized via decapitation. Pups aged Day 0 post partum up to and including Day 20 post partum will be euthanized via an intraperitoneal

injection of a sodium-pentobarbital-based euthanasia solution (150 mg/kg) followed by exsanguination.

Males:	Pups culled on Day 4 post partum: Day 4 post partum
	F0 and F1 males selected for dosing: Approximately 2 weeks after the end of the mating period (approximately 4-weeks following the last dose).
	F1 males not selected for dosing: After completion of animal selection for the dosing phase
	F2 males not selected for dosing: After completion of animal selection for the dosing phase
	F2 males selected for dosing: Approximately 24 hours following the last dose.
Females:	Pups culled on Day 4 post partum: Day 4 post partum
	F0 and females mated to F1 males producing litters: Following weaning of their litters on day 21 post partum and at the direction of the principal investigator. Fifteen days after the end of the mating period for females not found sperm positive which are palpated not pregnant and at the discretion of the principal investigator.
	F1 female pups: Day 21 post partum at weaning
	F2 females: Day 21 post partum at weaning

Table 1-5 Study design, animal allocation and doses for F0 animals

Group	Number/ Sex	Animal numbers*		Concentration (v/v)	Dose volume (mL/kg)
		males	females		males
1 Control	12	1001-1012	1501-1512	100% olive oil	0.5
2 CCl_4	12	2001-2012	2501-2512	50/50 CCl_4:olive oil	0.5

*Males only will be dosed; females will not be dosed

Table 1-6 Study design, animal allocation and doses for F1 animals

Group*	Number/ Sex	Animal numbers*		Concentration (v/v)	Dose volume (mL/kg)
		males	females		males
7 F0 control/ F1 control	12	7001-12	7501-12	100% olive oil	0.5
8 F0 control/ F1 treated	12	8001-12	8501-12	50/50 CCl_4:olive oil	0.5
9 F0 treated/ F1 control	12	9001-12	9501-12	100% olive oil	0.5
10 F0 treated/ F1 treated	12	10001-12	10501-12	50/50 CCl_4:olive oil	0.5

* Males only will be dosed; females will not be dosed

Table 1-7 Study design, animal allocation and doses for F2 animals

Group	Number	Animal numbers males	Concentration (v/v)	Dose volume (mL/kg) males
11 F0 control/ F1 control/ F2 control	10	11001-10	100% olive oil	0.5
12 F0 control/ F1 control/ F2 treated	10	12001-10	8/92 CCl_4:olive oil	0.5
13 F0 control/ F1 control/ F2 treated	10	13001-10	50/50 CCl_4:olive oil	0.5

14<br>F0 control/ F1 treated/ F2 control	10	14001-10	100% olive oil	0.5
15<br>F0 control/ F1 treated/ F2 treated	10	15001-10	8/92 CCl_4:olive oil	0,5
16<br>F0 control/ F1 treated/ F2 treated	10	16001-10	50/50 CCl_4:olive oil	0.5
17<br>F0 treated/ F1 control/ F2 control	10	17001-10	100% olive oil	0.5
18<br>F0 treated/ F1 control/ F2 treated	10	18001-10	8/92 CCl_4:olive oil	0.5
19<br>F0 treated/ F1 control/ F2 treated	10	19001-10	50/50 CCl_4:olive oil	0.5
20<br>F0 treated/ F1 treated/ F2 control	10	20001-10	100% olive oil	0.5
21<br>F0 treated/ F1 treated/ F2 treated	10	21001-10	8/92 CCl_4:olive oil	0.5
22<br>F0 treated/ F1 treated/ F2 treated	10	22001-10	50/50 CCl_4:olive oil	0.5

1.2.3 Administration of test and control articles

Route and frequency:	Orally, via gavage using glass/non-reactive polypropylene syringes, three times weekly for 6 weeks for F0, F1 and F2 males (i.e., days 1, 3, 5, 8, 10, 12, 15, 17, 19, 22, 24, 26, 29, 31, 33, 36, 38).
Justification for route:	The oral route has been used in previous studies of this type.

Dose volume:	0.5 mL/kg. Dose volumes will be based on the most recent body weight and will be calculated to two decimal places.
Administration method:	Test/control article formulations will be inverted gently and then stirred via a magnetic stir bar and plate during administration.

1.2.4 Study activities

Study activities will be performed as indicated in the study schedules below.

Veterinary treatments or other actions (including euthanasia) will be approved by the Principal Investigator/designee in consultation with a Veterinarian and documented in the raw data.

Table 1-8 Study schedule F0 generation

Major activities	Date, day, duration or study week
Animal arrival	Males – 22-Jan-2019<br>Females – 19-Mar-2019
Start of pretest	Males – 29-Jan-2019<br>Females – 25-Mar-2019
First day of dosing	Males – 04-Feb-2019 (day 1 of week 1)<br>Females – Will not be dosed
Mortality/moribundity checks	At least once daily (PM) on weekdays and at least once daily (AM) on weekends, holidays and plant emergencies/closings, unless noted otherwise.
Clinical observations	Pretest (males and females): At least once daily<br>Day 1 of dosing (males): Prior to dosing, and at 1 and 3 hours post dose.<br>Other dosing days (males) - prior to dosing and approximately 2 hours post-dose.<br>Non-dosing days (males) – once daily<br>Study period (females) – once daily<br>Additional observations may be obtained and will be documented in the raw data.
Body weights	Pretest (males and females): at least once<br>Dosing, recovery and mating (males): Twice weekly (*e.g.*, days 1, 4, 8, 11, 15, etc.) until termination, unless noted otherwise.<br>Study period (females) – not required<br>Additional weights may be obtained as part of a health check.
Food consumption	Males – calculated twice weekly (*e.g.*, days 4, 8, 11, 15, etc.) on body weight days until the initiation of mating.<br>Females – not required

Major activities	Date, day, duration or study week
	Additional intervals may be obtained as part of a health check. Animals will be fasted overnight for scheduled clinical pathology/necropsy, unless directed otherwise.
Urinalysis for potential biomarkers (all surviving males)	12-Mar-2019, day 37 following dose administration on day 36 and the week after completion of mating
Last day of dosing	13-Mar-2019 (day 38; groups 1 and 2)
Clinical biochemistry (all surviving males)	14-Mar-2019– day 39, approximately 24 hours after dosing on day 38, 28-Mar-2019 (Day 53) prior to the initiation of the mating period and prior to necropsy, unless noted otherwise.Additional samples will be collected if deemed necessary and when possible (*e.g.*, to monitor animal health). Samples will be collected at unscheduled euthanasias, when possible.
Potential biomarker/exploratory investigations (serum and plasma) (all surviving males)	14-Mar-2019 - day 39; approximately 24 hours after dosing on day 38 (all groups) and prior to necropsy (groups 1 and 2), unless noted otherwise. Samples will be collected at unscheduled euthanasias, when possible.
Start of 2-week recovery period (males)	15-Mar-2019
Initiation of co-housing (groups 1 and 2)	01-Apr-2019
First possible day 0 gestation	02-Apr-2019
End of mating	15-Apr-2019. The mating period may be extended at the discretion of the Principal Investigator and documented in the data file.
First expected deliveries	23-Apr-2019 (day 21 gestation)
Last expected deliveries	07-May-2019
First expected day 4 culls	27-Apr-2019
Last expected day 4 culls	11-May-2019
First expected day 21 post partum/F1 selections	14-May-2019
Last expected day 21 post partum/F1 selections	28-May-2019
Main study F0 male necropsies/sperm collection	18-Apr-2019
First potential day 21 post partum female and unselected pup necropsies	14-May-2019
Last potential day 21 post partum female and unselected pup necropsies	28-May-2019

Table 1-9 Study schedule F1 generation

Major activities	Date, day, duration or study week
First expected F1 deliveries	23-Apr-2019
First expected F1 day 21 post partum/weaning	14-May-2019

Major activities	Date, day, duration or study week
F1 male dose initiation	08-July-2019 (9-11 weeks of age)
Last F1 male dose	14-Aug-2019
Start of 2-week recovery period (males)	17-Aug-2019
Female arrival	19-Aug-2019
Female start of pretest	26-Aug-2019
Initiation of co-housing	03-Sep-2019
First possible day 0 gestation	04-Sep-2019
End of co-housing	17-Sep-2019
First expected F2 deliveries	25-Sep-2019
Last expected F2 deliveries	09-Oct-2019
First expected day 21 post partum/F2 selections	16-Oct-2019
Last expected day 21 post partum/F2 selections	30-Oct-2019
Mortality/moribundity checks	At least once daily (PM) on weekdays and at least once daily (AM) on weekends, holidays and plant emergencies/closings, unless noted otherwise.
Clinical observations	Day 0-21 post partum – daily as part of the litter check<br>Day 21 post partum until the initiation of dosing for males and until termination for females – once daily<br>Day 1 of dosing (males): Prior to dosing, and at 1 and 3 hours post dose. At least twice daily on other dosing days (prior to dosing and approximately 2 hours post-dose.<br>Non-dosing days males – once daily<br>Additional observations may be obtained and will be documented in the raw data.
Body weights	Prior to the initiation of dosing (males): Twice weekly beginning when all litters have been weaned.<br>Dosing, recovery and mating (males): Twice weekly (*e.g.*, days 1, 4, 8, 11, 15, etc.) until termination, unless noted otherwise.<br>Females – at least once during pretest<br>Additional weights may be obtained as part of a health check.
Food consumption	Males - calculated twice weekly beginning with the initiation of dosing on days of body weight until the initiation of mating.<br>Females – not required<br>Additional intervals may be obtained as part of a health check. Animals will be fasted overnight for scheduled clinical pathology/necropsy, unless directed otherwise.
Urinalysis for potential biomarkers	13-Aug-2019, day 37 following dose administration

Major activities	Date, day, duration or study week
	on day 36 and the week after completion of mating (males only)
Clinical biochemistry	15-Aug-2019 – day 39, males only; approximately 24 hours after dosing on day 38, 29-Aug-2019 (Day 53) prior to the initiation of the mating period and prior to necropsy on 19-Sep-2019 and 20-Sep-2019, unless noted otherwise. Additional samples will be collected if deemed necessary and when possible (*e.g.*, to monitor animal health). Samples will be collected at unscheduled euthanasias, when possible.
Potential biomarker/exploratory investigations (serum and plasma, all surviving dosed males)	15-Aug-2019 – day 39, males only; approximately 24 hours after dosing on day 38 and prior to scheduled necropsy, unless noted otherwise. Samples will be collected at unscheduled euthanasias, when possible.
Male necropsies/sperm collection	19-Sep-2019 and 20-Sep-2019
First potential day 21 post partum female/unselected pup necropsies	16-Oct-2019

Table 1-10 Study schedule F2 generation

Major activities	Date, day, duration or study week
First expected F2 deliveries	25-Sep-2019
First expected Day 4 culls	29-Sep-2019
First expected F2 day 21 post partum/weaning	16-Oct-2019
**F2 dose initiation (males)**	**See Appendix 2 (staggered starts; approximately 9-11 weeks of age**)
Mortality/moribundity checks	At least once daily (PM) on weekdays and at least once daily (AM) on weekends, holidays and plant emergencies/closings, unless noted otherwise.
Clinical observations	Day 0-21 post partum – daily as part of the litter check<br>Day 21 post partum until the initiation of dosing– once daily<br>Day 1 of dosing: Prior to dosing, and at 0.5, 1 and 3 hours post dose. At least twice daily on other dosing days (prior to dosing and approximately 2 hours post-dose).<br>Non-dosing days – once daily<br>Additional observations may be obtained and will be documented in the raw data.
Body weights	Prior to the initiation of dosing (males): Twice weekly beginning when all litters have been weaned.<br>Dosing (males): Twice weekly (*e.g.*, days 1, 4, 8, 11, 15, etc.) through termination, unless noted

Major activities	Date, day, duration or study week
	otherwise.<br>Female – not required<br>Additional weights may be obtained as part of a health check.
Food consumption	Calculated twice weekly beginning with the initiation of dosing on body weight days.<br>Additional intervals may be obtained as part of a health check. Animals will be fasted overnight for scheduled clinical pathology/necropsy, unless directed otherwise.
Urinalysis for potential biomarkers	07-Jan-2020, day 37 following dose administration on day 36.
Clinical biochemistry	09-Jan-2020 – day 39, males only; approximately 24 hours after dosing on day 38, unless noted otherwise. Additional samples will be collected if deemed necessary and when possible (*e.g.*, to monitor animal health). Samples will be collected at unscheduled euthanasias, when possible.
Potential biomarker/exploratory investigations (serum and plasma; all surviving males)	09-Jan-2020 – day 39, males only; approximately 24 hours after dosing on day 38 prior to necropsy, unless noted otherwise. Additional samples will be collected if deemed necessary and when possible (*e.g.*, to monitor animal health). Samples will be collected at unscheduled euthanasias, when possible.
**End of dosing necropsies/sperm collections***	**See Appendix 2**

*Animals will be necropsied approximately 24 hours after the last dose administraton.

1.2.5 Sampling

Animals will be anesthetized and blood will be sampled from animals fasted overnight (less than 24 hours).

Table 1-11 Sampling schedule

Biochemistry (Serum tube)	Genomics (RNA protect)	Potential Biomarkers/ exploratory investigations (serum tube)	Potential Biomarkers/ exploratory investigations (EDTA tube)	Urine
0.5 mL	0.5 mL	1 mL	0.5 mL	1 mL

Blood will be collected at the end of the dosing period (approximately 24 hours after dose administration all groups) and prior to necropsy (groups 1, 2, 7 through 10) and processed to serum or plasma within 30 minutes of collection. The resultant serum will be divided approximately equally into 3 aliquots, snap frozen and retained frozen at ≤ -60ºC. The resultant plasma will be divided approximately equally into 2 aliquots, snap frozen and

retained frozen at ≤ -60ºC. Urine samples will be collected, divided into 3 approximately equal aliquots and stored frozen at ≤ -60ºC.

1.2.6 Male epidydimal sperm collection at necropsy

The epididymis (left and right) will be isolated for the collection and isolation of sperm for scheduled euthanasia F0, F1 and F2 dosed males only (detailed protocol for collection and isolation of sperm via swim-out and swim-up available upon request). Samples will not be collected from unscheduled euthanasia dosed males or animals not selected for dosing. The resulting samples will be divided into four aliquots prior to snap freezing. The sperm aliquots will be stored and transferred to Biobanking Sample Management for possible analyses.

1.2.7 Male testicular sperm counts

The left testes will be collected, decapsulated, weighed and frozen at necropsy for sperm count evaluations.

1.2.8 Necropsy, tissue sampling, organ weights and histopathologic processing

F0, F1 and F2 males selected for dosing: Euthanasia and terminal blood collections will be performed per laboratory procedures. Necropsies will be performed with a recording of macroscopic abnormalities. Liver weights will be recorded at necropsy.

Representative tissue samples will be taken and processed to H&E-stained tissue sections from all animals. Controlled fixation will be performed for all tissues collected. Tissue(s) from unscheduled necropsies will be processed at the discretion of the study pathologist. Additional tissues may be collected and/or processed, and other stains or procedures (*e.g.*, TEM, Sirius red staining, images) may be used at the discretion of the study pathologist. Controlled fixation and storage of specimens will follow appropriate laboratory procedures, unless directed otherwise.

Pups culled on Day 4 and 21 post partum: Euthanized and discarded.

F0, F1 and F2 females, F1 and F2 males not selected for dosing (excluding animals culled at Day 4 and 21 post partum): Euthanasia will be performed per laboratory procedures. Necropsies will be performed with a recording of macroscopic abnormalities. Organ weights will not be recorded, no tissues will be retained and the carcasses will be discarded.

1.2.9 Microscopic examination

All tissue sections processed will be examined and assessed with all observations being recorded in the raw data. Decedent animals will be processed and examined at the request of the study pathologist.

1.3 Tissue sampling

Tissues will be collected per laboratory procedures, prepared & biobanked for possible analyses.

Kidney: Right collected for FFPE. Left collected for OCT (frozen) and snap-frozen sampling; timed fixation will be performed.

Epididymides: will be used for the collection/isolation of sperm (see 1.2.7)

Liver: One piece left lateral lobe and one piece median lobe for both FFPE and OCT (frozen); timed fixation will be performed. One piece left left lateral lobe for snap-frozen sampling.

Skin (inguinal): both FFPE and OCT (frozen); timed fixation will be performed; skin will be shaved prior to the collection of tissues for snap-frozen sampling.

Testis: Right of the first five animals/group (6 animals/group for F0 and F1) collected in Davidson fixative (simple immersion) for hematoxylin-PAS histology. Right of the last five animals/group (6 animals/group for F0 and F1) fixed in 4% PFA in PBS (with small volume of formalin injected on one pole of each testis) for approximately 24 hours followed by several rinses with 70% ethanol and stored at 4°C in 70% ethanol until processed. In the event of multiple early deaths, animals may be re-assigned. Left (all animals) collected, decapsulated weighed and frozen for subsequent sperm count.

1.4 Clinical pathology

Clinical Biochemistry parameters

Alanine Aminotransferase (ALT)	Globulins (GLOB)	Chloride (CL)
Alkaline Phosphatase (ALP)	Glucose (GLU)	Calcium (CA)
Aspartate Aminotransferase (AST)	Urea (UREA)	Inorganic Phosphorus (PHOS)
Total Bilirubin (TBIL)	Creatinine (CREAT)	Triglycerides (TRIG)
Total Protein (TP)	Sodium (NA)	Cholesterol (CHOL)
Albumin (ALB)	Potassium (K)	Albumin/Globulin Ratio (AGR)
Creatine Kinase (CK)	Magnesium (MG)	Bicarbonate (BICARB)
Direct Bilirubin (DBIL)*	Indirect Bilirubin (IBIL)*	

*measured only if total bilirubin in serum exceeds 5 μmol/L

Appendix 1
Detailed multigenerational pedigree tracing

Animal references of progeny

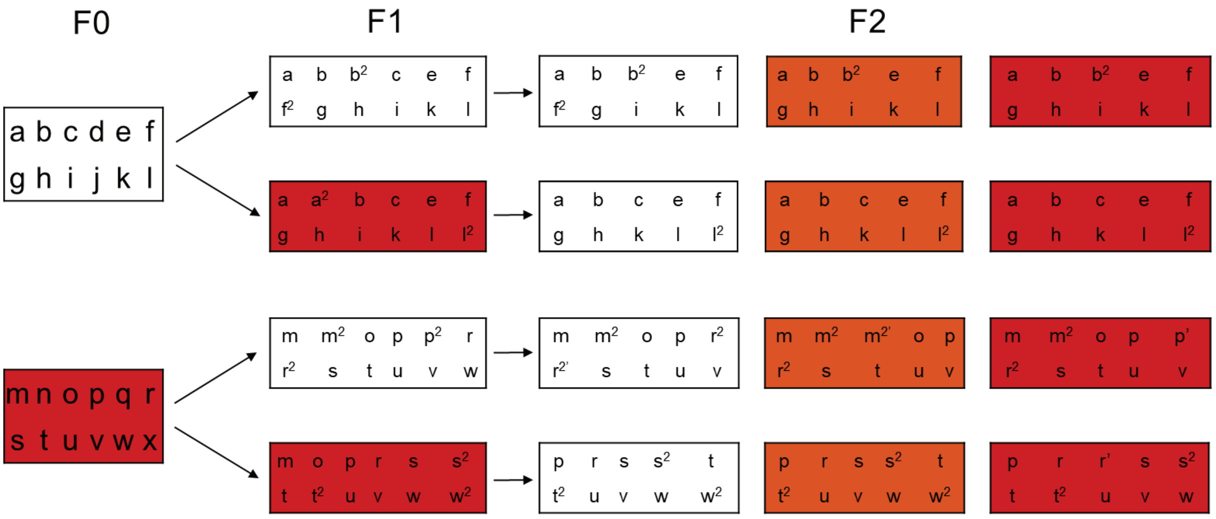

Schematic representation of pedigree tracing. Animal allocation in the different treatment groups illustrated with a letter for each F0 male and its corresponding offspring. If more than one pup was allocated to the same treatment group, the second one is marked with ² in F1 and ‘ in F2.

F1 Animal number progeny and litter tracing

F_0 Male Number	F_0 Dam Number	Litter Number	Chip Number	Pretest Number	F_1 Group	Animal Number
**1002**	1502	1	6E35756B3F	41	7	7001
			6E36665D60	40	8	8001
			6E31004A41	43	7	7002
			6E32306058	44		
			6E38203808	39		
			6E39443347	46		
			6E3704671A	45		
			6E39180F30	42		

F_0 Male Number	F_0 Dam Number	Litter Number	Chip Number	Pretest Number	F_1 Group	Animal Number
**1006**	1506	2	6E3875134F	49	7	7003
			6E313F7A6F	50	8	8002
			6E39691E63	51	7	7004
			6E337B4B2B	48		
			6E384B2824	47		
			-			
			-			
			-			

F_0 Male Number	F_0 Dam Number	Litter Number	Chip Number	Pretest Number	F_1 Group	Animal Number
**1012**	1512	3	6E35796F7E	53	7	7005
			6E3A055929	56	8	8004
			6E30596D65	55	8	8003
			6E365B4D2A	52		
			6E35621C25	58		
			6E372B3601	54		
			6E38441B66	57		
			-			

F_0 Male Number	F_0 Dam Number	Litter Number	Chip Number	Pretest Number	F_1 Group	Animal Number
**1001**	1501	4	6E39707C02	63	7	7006
			6E39601276	65	8	8006
			6E386B0913	61	8	8005
			6E397B1C5C	64		
			6E371D545F	66		
			6E33132336	59		
			6E39091C21	60		
			6E367A6930	62		

F_0 Male Number	F_0 Dam Number	Litter Number	Chip Number	Pretest Number	F_1 Group	Animal Number
**1003**	1503	5	6E356B4B6C	72	7	7007
			6E38640B0F	70	8	8007
			6E38792C22	67		
			6E3929063A	74		
			6E33425426	71		
			6E373A3932	73		
			6E354E4957	68		
			6E36253154	69		

F_0 Male Number	F_0 Dam Number	Litter Number	Chip Number	Pretest Number	F_1 Group	Animal Number
**1007**	1507	6	6E3837417A	76	7	7008
			6E356D6E3D	75	8	8008
			6E3803610E	79		
			6E3667523F	77		
			6E38393142	78		
			-			
			-			
			-			

F_0 Male Number	F_0 Dam Number	Litter Number	Chip Number	Pretest Number	F_1 Group	Animal Number
**1011**	1511	7	6E3853151D	82	7	7009
			6E37355312	83	8	8009
			6E386B5776	86		
			6E39466A42	81		
			6E39726669	85		
			6E102F7B37	87		
			6E37142315	80		
			6E30503F15	84		

F_0 Male Number	F_0 Dam Number	Litter Number	Chip Number	Pretest Number	F_1 Group	Animal Number
**1009**	1509	8	6E3818785D	92	7	7010
			6E355C4C65	93	8	8010
			6E35234411	90		
			6E31694232	95		
			6E381A7F37	91		
			6E370E581C	89		
			6E365E3049	94		
			6E353A6609	88		

F_0 Male Number	F_0 Dam Number	Litter Number	Chip Number	Pretest Number	F_1 Group	Animal Number
**1005**	1505	9	6E36630000	96	7	7011
			6E39261927	98	8	8011
			6E306B0E14	97		
			6E37096B09	378		
			-			
			-			
			-			
			-			

F_0 Male Number	F_0 Dam Number	Litter Number	Chip Number	Pretest Number	F_1 Group	Animal Number
**1008**	1508	10	6E3548681C	384	7	7012
			6E360E3B15	383	8	8012
			6E36671B23	385		
			6E33774102	380		
			6E11294852	386		
			6E38504264	382		
			6E35207960	381		
			6E386A4B4A	379		

F_0 Male Number	F_0 Dam Number	Litter Number	Chip Number	Pretest Number	F_1 Group	Animal Number
**2006**	2506	11	6E394E6649	390	9	9002
			6E3965794F	388	10	10001
			6E36592E20	389	9	9001
			6E363B1862	391		
			6E313B3472	387		
			6E3815544D	392		
			-			
			-			

F_0 Male Number	F_0 Dam Number	Litter Number	Chip Number	Pretest Number	F_1 Group	Animal Number
**2001**	2501	12	6E306B1844	395	9	9004
			6E2C187142	394	10	10002
			6E107D700C	393	9	9003
			-			
			-			
			-			
			-			
			-			

F_0 Male Number	F_0 Dam Number	Litter Number	Chip Number	Pretest Number	F_1 Group	Animal Number
**2004**	2504	13	6E374D5A3A	399	9	9006
			6E32363D4B	400	10	10003
			6E377C126A	398	9	9005
			6E36417832	397		
			6E37621F45	396		
			6E3825586A	401		
			6E37037A64	402		
			-			

F_0 Male Number	F_0 Dam Number	Litter Number	Chip Number	Pretest Number	F_1 Group	Animal Number
**2007**	2507	14	6E392E340C	408	9	9007
			6E3760571D	407	10	10005
			6E371A4353	404	10	10004
			6E37722526	406		
			6E373A3811	405		
			6E36705715	403		
			-			
			-			

F_0 Male Number	F_0 Dam Number	Litter Number	Chip Number	Pretest Number	F_1 Group	Animal Number
**2008**	2508	15	6E39471945	409	-	- (underweight)
			6E3566646B	416	9	9008
			6E36186044	412	10	10006
			6E366C4934	413	10	10007
			6E37594A24	411		
			6E377F3B7F	415		
			6E397A290C	410		
			6E39177679	414		

F_0 Male Number	F_0 Dam Number	Litter Number	Chip Number	Pretest Number	F_1 Group	Animal Number
**2011**	2511	16	6E36231859	419	9	9009
			6E36497217	420	10	10009
			6E356E5756	418	10	10008
			6E310E4E67	417		
			6E385F2002	421		
			-			
			-			
			-			

F_0 Male Number	F_0 Dam Number	Litter Number	Chip Number	Pretest Number	F_1 Group	Animal Number
**2009**	2509	17	6E312F7071	426	9	9010
			6E31665F18	423	10	10010
			6E1262220C	428		
			6E36246769	427		
			6E375A6212	425		
			6E37310848	422		
			6E387D300C	424		
			-			

F_0 Male Number	F_0 Dam Number	Litter Number	Chip Number	Pretest Number	F_1 Group	Animal Number
**2010**	2510	18	6E39123B67	429	9	9011
			6E31505B11	434	10	10011
			6E35334253	432		
			6E37097B77	430		
			6E366D6272	435		
			6E35661075	431		
			6E39545D39	436		
			6E2B241240	433		

F_0 Male Number	F_0 Dam Number	Litter Number	Chip Number	Pretest Number	F_1 Group	Animal Number
**2003**	2503	19	6E354E6112	437	9	9012
			6E3560430E	443	10	10012
			6E104D1623	439		
			6E391B1A04	444		
			6E36416C56	442		
			6E32747746	441		
			6E321B3C30	438		
			6E3562407E	440		

F2 Animal number progeny and litter tracing

F_1 Male Number	F_1 Dam Number	Litter Number	Chip Number	Pretest Number	F_2 Group	Animal Number
**7008**	7508	-	6E3B5D184D	132	11	11001
			6E380D1D6A	134	12	12001
			6E3A12205B	131	13	13001
			6E3E332C58	135		
			6E3C014221	137		
			6E3D066968	133		
			6E3D370C44	136		
			-			

F_1 Male Number	F_1 Dam Number	Litter Number	Chip Number	Pretest Number	F_2 Group	Animal Number
**7009**	7509	-	6E396A6B2D	143	11	11002
			6E3D275F38	144	12	12002
			6E3C200475	141	13	13002
			6E3756383C	139		
			6E370E4210	142		
			6E36545F48	140		
			6E3A577211	138		
			-			

F_1 Male Number	F_1 Dam Number	Litter Number	Chip Number	Pretest Number	F_2 Group	Animal Number
**7010**	7510	-	6E3B23075E	150	11	11003
			6E3E6B2C02	152	12	12003
			6E3B643030	146	13	13003
			6E3E0F2865	145		
			6E3A7B0F26	149		
			6E3B414A49	148		
			6E37333E7C	151		

			6E38762C04	147		

F_1 Male Number	F_1 Dam Number	Litter Number	Chip Number	Pretest Number	F_2 Group	Animal Number
**7001**	7501	-	6E3D280446	99	11	11004
			6E39095127	100	12	12004
			6E3E265D0B	101	13	13004
			-			
			-			
			-			
			-			
			-			

F_1 Male Number	F_1 Dam Number	Litter Number	Chip Number	Pretest Number	F_2 Group	Animal Number
**7002**	7502	-	6E3E302C2B	107	11	11005
			6E3E0F605D	102	12	12005
			6E38750F60	109	13	13005
			6E370F442A	106		
			6E3673790D	104		
			6E397C115C	108		
			6E3B4F536E	103		
			6E3B0C0555	105		

F_1 Male Number	F_1 Dam Number	Litter Number	Chip Number	Pretest Number	F_2 Group	Animal Number
**7003**	7503	-	6E374A0C6C	113	11	11006
			6E3B56433F	110	12	12006
			6E38174A3C	111	13	13006
			6E36600A01	112		
			-			
			-			
			-			
			-			

F_1 Male Number	F_1 Dam Number	Litter Number	Chip Number	Pretest Number	F_2 Group	Animal Number
**7004**	7504	-	6E39735E46	114	11	11007

F_1 Male Number	F_1 Dam Number	Litter Number	Chip Number	Pretest Number	F_2 Group	Animal Number
**7005**	7505	-	6E3D193F05	120	11	11008
			6E3B667339	115	12	12007
			6E36080B03	117	13	13007
			6E3A62687E	118		
			6E3E3B0F1B	116		
			6E370C6C1C	121		
			6E3E4F2F0E	119		
			6E372C6340	122		

F_1 Male Number	F_1 Dam Number	Litter Number	Chip Number	Pretest Number	F_2 Group	Animal Number
**7006**	7506	-	6E3A552920	123	11	11009
			6E3B616516	130	12	12008
			6E3D230078	128	13	13008
			6E3E490A5F	127		
			6E3E303627	126		
			6E390D0536	125		
			6E3C69150A	129		
			6E39392776	124		

F_1 Male Number	F_1 Dam Number	Litter Number	Chip Number	Pretest Number	F_2 Group	Animal Number
**7011**	7511	-	6E3B7B446A	155	11	11010
			6E3E2F7034	154	12	12009
			6E3760240E	156	13	13009
			6E3643252D	157		
			6E3E5A0E5A	153		
			-			
			-			
			-			

F_1 Male Number	F_1 Dam Number	Litter Number	Chip Number	Pretest Number	F_2 Group	Animal Number
**7012**	7512	-	6E363A6200	163	12	12010
			6E3C3C6022	162	13	13010
			6E396E3077	161		
			6E3B1C6654	160		
			6E3760182F	164		
			6E3669480F	159		
			6E363E3328	158		
			-			

F_1 Male Number	F_1 Dam Number	Litter Number	Chip Number	Pretest Number	F_2 Group	Animal Number
**8001**	8501	-	6E34535B5A	223	14	14001
			6E38267A2E	224	15	15001
			6E380A5130	225		
			6E384F652E	221	16	16001
			6E3958664E	226		
			6E39163119	220		
			6E380B2F48	222		
			6E3B355267	219		

F_1 Male Number	F_1 Dam Number	Litter Number	Chip Number	Pretest Number	F_2 Group	Animal Number
**8002**	8502	-	6E361D5944	228	14	14002
			6E3D6C273F	231	15	15002
			6E3C6B0B7B	230	16	16002
			6E3B0F2979	232		
			6E3B737757	227		
			6E38405D3D	233		
			6E3A434422	234		
			6E342F6D48	229		

F_1 Male Number	F_1 Dam Number	Litter Number	Chip Number	Pretest Number	F_2 Group	Animal Number
**8003**	8503	-	6E3A782407	237	14	14003
			6E3C690928	236	15	15003
			6E392A6407	235	16	16003
			6E38177875	238		
			-			
			-			
			-			
			-			

F_1 Male Number	F_1 Dam Number	Litter Number	Chip Number	Pretest Number	F_2 Group	Animal Number
**8004**	8504	-	6E3D2E4B1F	244	14	14004
			6E3C62590B	241	15	15004
			6E116A0F34	243	16	16004
			6E3B7C184E	239		
			6E362F7F75	245		
			6E376A2E4C	242		
			6E3B3B714F	246		
			6E3D3A0811	240		

F_1 Male Number	F_1 Dam Number	Litter Number	Chip Number	Pretest Number	F_2 Group	Animal Number
**8005**	8505	-	6E377D2203	247	14	14005
			6E372A2271	251	15	15005
			6E38417176	250	16	16005
			6E3A65174E	249		
			6E3E480423	248		
			-			
			-			
			-			

F_1 Male Number	F_1 Dam Number	Litter Number	Chip Number	Pretest Number	F_2 Group	Animal Number
**8007**	8507	-	6E3A3E4C31	252	14	14006
			6E3C785F79	253	15	15006
			6E3E076707	254	16	16006
			6E37116257	257		
			6E3A440B6F	255		
			6E3D1B4E45	258		
			6E3A135F00	256		
			-			

F_1 Male Number	F_1 Dam Number	Litter Number	Chip Number	Pretest Number	F_2 Group	Animal Number
**8008**	8508	-	6E357B4172	259	14	14007
			6E3B53204A	263	15	15007
			6E39334B60	260	16	16007
			6E3B7D7903	265		
			6E360C6E4C	261		
			6E396A6466	262		
			6E3D5D4609	264		
			-			

F_1 Male Number	F_1 Dam Number	Litter Number	Chip Number	Pretest Number	F_2 Group	Animal Number
**8009**	8509	-	6E3C18303E	266	14	14008
			6E3D02641B	267	15	15008
			6E3C4A7514	268	16	16008
			-			
			-			
			-			
			-			
			-			

F_1 Male Number	F_1 Dam Number	Litter Number	Chip Number	Pretest Number	F_2 Group	Animal Number
**8011**	8511	-	6E38294E61	271	14	14009
			6E3B3D436D	273	15	15009
			6E3C376232	275	16	16009
			6E3D48086C	274		
			6E3B46520E	272		
			6E3E465D7D	269		
			6E373E0E4E	270		
			6E37746A7F	276		

F_1 Male Number	F_1 Dam Number	Litter Number	Chip Number	Pretest Number	F_2 Group	Animal Number
**8012**	8512	-	6E3A136036	283	14	14010
			6E3B6F2F21	280	15	15010
			6E374A2110	279	16	16010
			6E3D210631	281		
			6E3B777C0A	282		
			6E370D1F1B	278		
			6E3D126259	277		
			-			

F_1 Male Number	F_1 Dam Number	Litter Number	Chip Number	Pretest Number	F_2 Group	Animal Number
**9002**	9502	-	6E3A662928	169	17	17001
			6E3A6E4826	165	18	18001
			6E387F114B	168	19	19001
			6E3D137D78	167	17	17002
			6E3A4E784C	166		
			-			
			-			
			-			

F_1 Male Number	F_1 Dam Number	Litter Number	Chip Number	Pretest Number	F_2 Group	Animal Number
**9003**	9503	-	6E3D64113B	172	17	17003
			6E3E387861	170	18	18002
			6E3D5E4759	171	19	19002
			-			
			-			
			-			
			-			
			-			

F_1 Male Number	F_1 Dam Number	Litter Number	Chip Number	Pretest Number	F_2 Group	Animal Number
**9004**	9504	-	6E3725206D	176	17	17004
			6E3C443765	179	18	18003
			6E38172D4B	175	19	19003
			6E377E6C61	177	18	18004
			6E3962575B	178		
			6E37401F35	173		
			6E3A662740	174		
			-			

F_1 Male Number	F_1 Dam Number	Litter Number	Chip Number	Pretest Number	F_2 Group	Animal Number
**9005**	9505	-	6E3C526B39	182	17	17005
			6E3E002932	183	18	18005
			6E3B43060B	185	19	19004
			6E3A462563	181	19	19005
			6E3742601D	180		
			6E37087A40	184		
			-			
			-			

F_1 Male Number	F_1 Dam Number	Litter Number	Chip Number	Pretest Number	F_2 Group	Animal Number
**9007**	9507	-	6E3908614E	191	17	17006
			6E32536C39	192	18	18006
			6E3B455A3B	188	19	19006
			6E3C346241	186		
			6E35464C39	187		
			6E37020006	190		
			6E3D4D0032	189		
			-			

F_1 Male Number	F_1 Dam Number	Litter Number	Chip Number	Pretest Number	F_2 Group	Animal Number
**9008**	9508		6E3D2C6E42	194	17	17007
			6E3B2C7772	197	18	18007
			6E352F3260	196	19	19007
			6E3E5D2B1F	195		
			6E3D3E6C7D	198		
			6E36723A77	193		
			-			
			-			

F_1 Male Number	F_1 Dam Number	Litter Number	Chip Number	Pretest Number	F_2 Group	Animal Number
**9010**	9510	-	6E3C36140C	202	17	17008
			6E3E007532	201	18	18008
			6E383A3977	203	19	19008
			6E39212B11	200		
			6E3D733E60	199		
			-			
			-			
			-			

F_1 Male Number	F_1 Dam Number	Litter Number	Chip Number	Pretest Number	F_2 Group	Animal Number
**9011**	9511	-	6E3D611846	207	17	17009
			6E3A04093A	210	18	18009
			6E386C6B22	204	19	19009
			6E36480875	208		
			6E381C482C	211		
			6E3E2A6052	209		
			6E36432C48	206		
			6E39301920	205		

F_1 Male Number	F_1 Dam Number	Litter Number	Chip Number	Pretest Number	F_2 Group	Animal Number
**9012**	9512	-	6E38630D5E	212	17	17010
			6E3E574961	217	18	18010
			6E3C606074	213	19	19010
			6E396F0E76	215		
			6E3665006B	216		
			6E3A167173	214		
			6D7E67242F	218		
			-			

F_1 Male Number	F_1 Dam Number	Litter Number	Chip Number	Pretest Number	F_2 Group	Animal Number
**10001**	10501	-	6E3C4A2727	291	20	20001
			6E3906002B	286	21	21001
			6E3C464532	284	22	22001
			6E3B715329	287	22	22002
			6E3A0C1B27	290		
			6E373C2927	288		
			6E3E644D73	285		
			6E387E262F	289		

F_1 Male Number	F_1 Dam Number	Litter Number	Chip Number	Pretest Number	F_2 Group	Animal Number
**10002**	10502	-	6E3C480E26			
			6E3A006C75			
			6E385D5376			
			6E367E4440			
			6E3755484B			
			6E3C446556			
			-			
			-			

F_1 Male Number	F_1 Dam Number	Litter Number	Chip Number	Pretest Number	F_2 Group	Animal Number
**10003**	10503	-	6E3A15634A	294	20	20002
			6E13233263	292	21	21002
			6E35653B54	293	22	22003
			-			
			-			
			-			
			-			
			-			

F1 Male Number	F1 Dam Number	Litter Number	Chip Number	Pretest Number	F2 Group	Animal Number
**10004**	10504	-	6E3B581976	297	20	20003
			6E3C3E5038	298	21	21003
			6E3B66502D	295	22	22004
			6E35362A77	296		
			6E3B123807	445		
			6E372A2E2D	446		
			-			
			-			

F1 Male Number	F1 Dam Number	Litter Number	Chip Number	Pretest Number	F2 Group	Animal Number
**10005**	10505	-	6E386C654A	447	20	20004
			6E3B7E0011	448	21	21004
			6E3C3E2426	451	22	22005
			6E3C4D2342	450		
			6E3C704E73	449		
			-			
			-			
			-			

F1 Male Number	F1 Dam Number	Litter Number	Chip Number	Pretest Number	F2 Group	Animal Number
**10006**	10506	-	6E3B797553	456	20	20005
			6E364B7D3B	453	21	21005
			6E3D7D1B66	454	22	22006
			6E3C0E7040	452		
			6E3C72336F	455		
			-			
			-			
			-			

F1 Male Number	F1 Dam Number	Litter Number	Chip Number	Pretest Number	F2 Group	Animal Number
**10007**	10507	-	6E391F107A	458	20	20006
			6E397A463D	459	21	21006
			6E3D5D4213	457	22	22007
			-			
			-			
			-			
			-			
			-			

F1 Male Number	F1 Dam Number	Litter Number	Chip Number	Pretest Number	F2 Group	Animal Number
**10008**	10508	-	6E3D7D0453	464	20	20007
			6E373D1F60	465	21	21007
			6E39267C64	460	22	22008
			6E3D266634	462		
			6E370A302C	463		
			6E39220C6C	466		
			6E3B2C6C5D	461		
			-			

F_1 Male Number	F_1 Dam Number	Litter Number	Chip Number	Pretest Number	F_2 Group	Animal Number
**10009**	10509		6E39633B69	468	20	20008
			6E38402923	467	21	21008
			-			
			-			
			-			
			-			
			-			
			-			

F_1 Male Number	F_1 Dam Number	Litter Number	Chip Number	Pretest Number	F_2 Group	Animal Number
**10010**	10510	-	6E36113B51	469	20	20009
			6E39106175	471	21	21009
			6E3E50391A	472	22	22009
			6E3B452F06	476		
			6E3A082A75	473		
			6E381D4D01	475		
			6E3B156423	470		
			6E3A122D24	474		

F_1 Male Number	F_1 Dam Number	Litter Number	Chip Number	Pretest Number	F_2 Group	Animal Number
**10011**	10511	-	6E366E4F7D	483	20	20010
			6E393A6245	482	21	21010
			6E3C590321	477	22	22010
			6E37475B29	479		
			6E37450B49	478		
			6E374B772D	481		
			6E366A5749	480		
			6E3C277B45	484		

Appendix 2

Staggering and randomization scheme for F2 dose-response treatment

Groups (animals)	Treatment Day 1	Days of dosing (17 doses)	Day of necropsy (24 hours post last dose)
11 (11001-11003)<br>12(12001-12003)<br>13 (13001-13003)<br>14 (14001-14003)<br>15(15001-15003)<br>16 (16001-16003)<br>17 (17001-17002)<br>18(18001-18002)<br>19 (19001-19002)<br>20 (20001-20002)<br>21(21001-21002)<br>22 (22001-22002)	Monday December 2	December 2, 4, 6, 9, 11, 13, 16, 18, 20, 23, 25, 27, 30 Jan 1, 3, 6 and 8	January 9 (Day 39)
11 (11004-11005)<br>12(12004-12005)<br>13 (13004-13005)<br>14 (14004-14005)<br>15(15004-15005)<br>16 (16004-16005)<br>17 (17003-17005)<br>18(18003-18005)<br>19 (19003-19005)<br>20 (20003-20005)<br>21(21003-21005)<br>22 (22003-22005)	Monday December 9	December 9, 11, 13, 16, 18, 20, 23, 25, 27, 30 Jan 1, 3, 6, 8, 10,13 and 15	January 16 (Day 39)
11 (11006-11008)<br>12(12006-12008)<br>13 (13006-13008)<br>14 (14006-14008)<br>15(15006-15008)<br>16 (16006-16008)<br>17 (17006-17007)<br>18(18006-18007)<br>19 (19006-19007)<br>20 (20006-20007)<br>21(21006-21007)<br>22 (22006-22007)	Monday December 16	December 16, 18, 20, 23, 25, 27, 30 Jan 1, 3, 6, 8, 10, 13,15, 17, 20 and 22	January 23 (Day 39)
11 (11009-11010)<br>12(12009-12010)<br>13 (13009-13010)<br>14 (14009-14010)<br>15(15009-15010)<br>16 (16009-16010)<br>17 (17008-17010)<br>18(18008-18010)<br>19 (19008-19010)<br>20 (20008-20010)<br>21(21008-21010)<br>22 (22008-22010)	Monday December 23	December 23, 25, 27, 30 Jan 1, 3, 6, 8, 10, 13, 15, 17, 20, 22, 24, 27 and 29	January 30 (Day 39)
