# Supplementary material for: Unaltered hepatic wound healing response in male rats with ancestral liver injury
Source: Nat Commun. 2023 Oct 10;14:6353. doi: 10.1038/s41467-023-41998-w (PMC10564731; doi:10.1038/s41467-023-41998-w)
Supplement: Supplementary file 3 — Description of Additional Supplementary Files [file 41467_2023_41998_MOESM3_ESM.pdf]

## Description of Additional Supplementary Files

**Supplementary Data 1.** Clinical observations, clinical pathology and pathology

This document gives a detailed description of clinical findings (mortality, clinical observations, body weight, food consumption in F0-F2), clinical pathology (F0-F2) and microscopic histo-pathology evaluation (F2). This data extends on the data presented in Fig. 1 (F0, F1 generations) and Fig. 2 (F2 generation).

**Supplementary Data 2.** Gene lists and GO term analysis of the various gene sets under ancestral liver injury influence

- Tab '*1523 genes F0'*: The 1523 genes whose liver expression correlated with F0 treatment history: For each of the 1523 genes, a subset of DESeq2 statistics is provided as follows. From the model with design ~F0 + F1 + F2, the estimated base mean expression of the gene (column "baseMean"), as well as the estimated log2 fold-changes contributed via F0 (column "F0_log2FC") and F1 (column "F1_log2FC") treatment history and the corresponding FDR of the effect sizes ("F0_FDR" and "F1_FDR") are provided. From the model with design ~group, the estimated log2 fold-changes and FDR for the contrast of animal group A vs C (columns "AvsC_log2FC" and "AvsC_FDR", respectively) and for the contrast of animal group A vs D (columns "AvsD_log2FC" and "AvsD_FDR", respectively) are provided.
- Tab '*1523 genes GO F0_SuppFig5'*: GO term over-representation analysis of the 1523 gene set: This table lists the summary statistics of the Gene Ontology (GO) term over-representation analysis of the 1523 gene set for all GO terms achieving cutoffs of p-value < 0.1 and q-value < 0.2. For each GO term hierarchy (column "ONTOLOGY"), the GO term ID and description are provided. The column "GeneRatio", gives the ratio of the number of F0-assocated genes (also in column "count) over the number of GO-term annotated genes. The column "BgRatio" gives the number of GO-term annotated genes among all genes annotated in the hierarchy. The gene names of genes overlapping with the GO-term are provided in column "geneID". The remaining columns provide the different significance quantifications. This table was used to build the representation in Supplementary Fig 5.
- Tab '*69 genes F0_F1'*: 69 genes whose liver expression correlated with F0+F1 cumulative treatment history: A subset of Tab '*1523 genes F0'*. Selection of the 69 genes as described in the main text.
- Tab '*69 genes GO F0_F1_Fig5d'*: GO term over-representation analysis of 69 gene set: This table lists the summary statistics of the Gene Ontology (GO) term over-representation analysis of the 69 gene set for all GO terms achieving cutoffs of p-value < 0.1 and q-value < 0.2. Same format as Tab '*1523 genes GO F0_SuppFig5'*. The table was used to build the representation in Fig. 5d.
- Tab '*66 genes F0_kidney'*: 66 genes whose kidney expression correlated with F0 treatment history: For each of the 66 genes, a subset of DESeq2 statistics is provided in the same format as for Tab '*1523 genes F0'*.

**Supplementary Data 3.** Table for all study biochemistry data

This document provides the full panel of values obtained for the complete set of 26 clinical pathology and biochemistry markers assessed across all treatment groups of the F0, F1 and F2 generations. The data was obtained as described in Supplementary Methods and Supplementary Data 1.
