# Supplementary material for: Unaltered hepatic wound healing response in male rats with ancestral liver injury
Source: Nat Commun. 2023 Oct 10;14:6353. doi: 10.1038/s41467-023-41998-w (PMC10564731; doi:10.1038/s41467-023-41998-w)
Supplement: Supplementary file 4 — Supplementary Data 1 [file 41467_2023_41998_MOESM4_ESM.pdf]

Supplementary Data 1

This document gives a detailed description of clinical findings (mortality, clinical observations, body weight, food consumption in F0-F2), clinical pathology (F0-F2) and microscopic histopathology evaluation (F2). This data extends the data presented in Fig. 1 (F0, F1 generations) as well as Fig. 2 and Fig. 3 (F2 generation).

A three times weekly carbon tetrachloride (CCl_4) oral (gavage) multi-generational investigational pharmacology and toxicology study in Sprague Dawley rats

Clinical observations, clinical pathology and pathology

1 Clinical observations, clinical pathology and pathology

1.1 F0 generation

1.1.1 Clinical findings, body weight, food consumption

Mortality

Animal no. 2005 was found dead on day 41 (following 18 50% CCl_4 dose administrations). Prior to its death, this animal had a pale appearance, decreased motor activity, chromorhinorrhea, salivation and piloerection. Necropsy revealed fluid in the abdominal cavity. All other animals survived until scheduled necropsy.

Clinical Observations

Clinical signs of potential toxicity associated with administration of CCl_4 were observed in the majority of animals after approximately 4 weeks of three times weekly dosing and included salivation, piloerection, muzzle staining and/or decreased motor activity.

Body Weight

For animals administered 50% CCl_4 (group 2), mean body weight gains were statistically reduced compared to the control group (9-45%) for the entire dosing period. There was improvement over the course of the treatment period with the most significant reductions compared to the control occurring in the first 2 weeks of dosing. The reduction in gains led to statistically reduced (up to 10%) mean body weights compared to the control group during the dosing period, however, at the end of the dosing period mean body weight was only reduced 1.6% compared to the control.

Food Consumption

For animals in group 2 administered 50% CCl_4, food consumption was statistically reduced (3-22%) compared to the control group at most intervals during the first 5 weeks of dosing. Toward the end of the dosing period, there appeared to be a rebound phenomenon with statistically significant increases (12-19%) in food consumption compared to the control group.

1.1.2 Clinical Pathology

Mean change of group 2 was compared to mean change of group 1 to determine and quantify compound related changes.

Compound related changes were seen starting on day 39 and included changes that were suggestive of hepatobiliary perturbation (minimal to marked increase in alanine aminotransferase, aspartate aminotransferase, alkaline phosphatase and bilirubin due to increase in both, direct and indirect bilirubin). The enzymes activities were with highest magnitude on day 39, compared to control, and decreased towards control values until day 74. Additional changes seen on day 39 included minimal increase in urea, and decrease in globulins, albumins and total protein

On day 74, animal no. 2004 had mild increase in alanine aminotransferase, aspartate aminotransferase, total bilirubin, cholesterol and decrease in albumin.

Table 1-1 Changes seen in F0 rats, administered 50/50 CCl_4 and oil

Parameters	Day	50/50 CCl_4 / oil
**Aspartate aminotransferase**	**39**	+4132%
(AST)	**53**	+39%
	**74**	+49%
**Alanine aminotransferase**	**39**	+6430%
(ALT)	**53**	+101%
	**74**	+100%
**Alkaline phosphatase**	**39**	+293%
(ALP)	**53**	+63%
	**74**	+34%
**Total Bilirubin**	**39**	+1823%
(BILI)	**53**	+69%
	**74**	+62%
**Total protein**	**39**	-9%
(TP)	**53**	-8%
	**74**	-
**Albumin**	**39**	-7%
(ALB)	**53**	-11%
	**74**	-
**Globulin**	**39**	-12%
(GLOB)	**53**	-
	**74**	-
**Urea**	**39**	+35%
(UREA)	**53**	+6%
	**74**	+9%

Day 39: sampling at peak fibrosis; day 53: sampling after 2 weeks recovery; day 74: sampling at necropsy

1.2 F1 generation

1.2.1 Clinical findings, body weight, food consumption

Mortality

All animals survived until scheduled necropsy.

Clinical Observations

Clinical signs considered related to administration to 50% CCl_4 were observed in the latter portion of the dosing period for animals in groups 8 and 10 (generally after 3 weeks) and included isolated

incidences of chromorhinorrhea, muzzle staining, piloerection, salivation and unkempt fur. Clinical signs occurred with similar incidence and frequency in groups 8 and 10. All other clinical signs noted were considered incidental to treatment with CCl_4.

Body Weight

Body weight loss (relative to dosing day 1) occurred in the majority of group 8 and 10 animals (50% CCl_4) between days 1-4 and in individual animals through day 43. Mean body weight gain was significantly reduced (-8 to -12%) in the group 8 and 10 animals compared the true vehicle control group (group 7) at all intervals through day 53. There were no clear differences between groups 8 and 10. For group 9 (administered the vehicle, male parents (F0) administered 50% CCl_4) mean body weight gain was comparable to the concurrent true vehicle control (group 7).

Food Consumption

Mean food consumption was reduced/statistically reduced through day 39 of the dosing period for animals in group 8 (-4 to -29%) and group 10 (-5 to -20%) compared to the true vehicle control group (group 7). For both groups 8 and 10, animals rebounded and were eating significantly more than the control group the remainder of the dosing period. There were no clear differences between groups 8 and 10. For group 9 (administered the vehicle, male parents (F0) administered 50% CCl_4) food consumption was comparable to the concurrent true vehicle control (group 7) throughout the dosing period.

1.2.2 Clinical Pathology

Compound related changes seen on day 39, included changes suggestive of hepatobiliary perturbation (marked increase in alanine aminotransferase and aspartate aminotransferase, moderate increase in alkaline phosphatase and total bilirubin, both due to direct and indirect bilirubin). Additional changes include minimal to mild decrease in globulin, total protein cholesterol and mild increase in urea. On day 74 all parameters were comparable to control.

No clear F0 treatment related changes were seen when comparing group 9 vs 7 or group 10 vs 8.

Table 1-2 Changes seen in F1 rats, administered 50/50 CCl_4 and oil

Parameters	Day	Group 8	Group 10
**Aspartate aminotransferase**	**39**	+2472%	+3871%
(AST)	**53**	+44%	+14%
	**74**	-	-
**Alanine aminotransferase**	**39**	+5487%	+7076%
(ALT)	**53**	+93%	+63%
	**74**	+55%	-
**Alkaline phosphatase**	**39**	+294%	+276%
(ALP)	**53**	+25%	+39%
	**74**	-	-

Parameters	Day	Group 8	Group 10
**Total Bilirubin**	**39**	+702%	+649%
(BILI)	**53**	+25%	-
	**74**	-	-
**Total protein**	**39**	-6%	-8%
(TP)	**53**	-4%	-3%
	**74**	-	-
**Globulin**	**39**	-13%	-13%
(GLOB)	**53**	-	-5%
	**74**	-	-
**Cholesterol**	**39**	-16%	-26%
(CHOL)	**53**	-	-
	**74**	-	-
**Urea**	**39**	+53%	+55%
(UREA)	**53**	-	+5%
	**74**	-	-

1.3 F2 generation

1.3.1 Clinical findings, body weight, food consumption

Mortality

Animal no. 17005 (F2 Vehicle Control/F1 Vehicle Control/F0 50% CCl_4) was euthanized moribund on day 30. Prior to euthanasia this animal was gasping and had chromodacryorrhea, decreased motor activity and muzzle swelling. Animal 19003 (F2 50% CCl_4/F1 Vehicle Control/ F0 50% CCl_4) was found dead on day 8 (4 dose administrations) immediately post dose there were no clinical signs prior to death. The cause of death in this animal was considered to be associated with laceration of liver. All remaining animals survived until scheduled necropsy.

Clinical Observations

There were no differentiating clinical signs in the F2 vehicle control groups that were progeny of F0 or F1 generation parents administered CCl_4 (groups 14, 17 and 20) compared to the true control group (group 11). For groups administered 8% CCl_4 (groups 12, 15, 18 and 21) salivation was noted on one or more occurrences for multiple in animals in each group and one rat in group 15 had piloerection on one day. For groups administered 50% CCl_4 (groups 13, 16, 19 and 22) there was a slight increase in the frequency of salivation noted compared to the low dose of CCl4. In addition, one rat in group 13 (animal no. 13004) appeared thin and had chromorhinorrhea, staining around the mouth and muzzle and piloerection noted on multiple days in the latter portion of the dosing period (day 23 and later). In addition, animal no. 13002 had decreased motor activity on day 35 and rat no. 13006 had urine with apparent blood on day 29. One rat in group 22 (animal no. 22003) had apparent blood in the urine on day 36. There were no clear differences between the

groups based on whether the F0 and/or F1 generations were administered the vehicle or CCl_4 and all other clinical signs noted in these groups were considered incidental to F2 generation administration of CCl_4.

Body Weight

Dose-responsive effects occurred in mean body weight gains and mean body weight. Slight body weight loss occurred in a few animals in groups 12, 15, 18 and 21 (8% CCl_4) from day 4 (relative to dosing day 1). Body weight gain (relative to dosing day 1) was slightly reduced compared to the true vehicle control (group 11) throughout the dosing period and on day 39 mean body weight gain was reduced 16, 24, 22 and 20% compared to the true vehicle control in groups 12, 15, 18 and 21, respectively. Mean body weights were slightly reduced compared to the true control group (2.5-6%) on day 39 for the animals in groups 12, 15, 18 and 21. For animals administered the 50% CCl_4 dose (groups 13, 16, 19 and 22), body weight loss occurred in the majority of animals on days 1-4 and mean body weight gains were significantly reduced compared to the true vehicle control throughout the dosing period (26-92%). On day 39, mean body weight was reduced compared to the true vehicle control group 11-16%, for groups 13, 16, 19 and 22. There were no clear differences between the treated groups at either CCl_4 dose attributable to the treatment of the prior generations. There were no clear differences in mean body weight gain or mean body weights between the true vehicle control group and groups 14, 17 and 20 (F2 generation administered the vehicle; F0 and/or F1 generation administered 50% CCl_4).

Food Consumption

Food consumption was reduced in a dose-dependent manner associated with the dose-dependent reduction in body weight parameters as indicated above. For animals administered the 8% CCl_4 dose, food consumption was significantly reduced (11-14%) compared to the true vehicle control group (group 11) on day 4 for groups 12, 15 and 21 and reduced (-9%) for group 18. In general, food consumption remained slightly reduced compared to the control throughout the dosing period (up to 12%) with the exception of day 25 for group 15 when food consumption was significantly reduced compared to the control group (14%). For animals administered the 50% CCl_4 (groups 13, 16, 19 and 22) food consumption was significantly reduced (19-30%) throughout the dosing period compared to the true vehicle control group. The reductions were significant on days 11, 25 and/or or 32 (up to 36% reduced compared to the control). There were no clear differences in the CCl_4-treated animals attributable to the treatment in prior generations and food consumption for the control groups with prior CCl_4 exposure in the F0 and/or F1 generations (groups 14, 17 and 20) was comparable to the true vehicle control.

1.3.2 Clinical Pathology

Dose related response (comparing group 12 & 13 vs 11, 15 & 16 vs 14, 18 & 19 vs 17, 21 & 22 vs 20): In all groups, compound related changes were dose related and included marked increase in alanine aminotransferase and aspartate aminotransferase, moderate increase in alkaline phosphatase and total bilirubin (both direct and indirect) and minimal increase in urea. In addition, in the high dose groups there was decrease in glucose and increase in creatinine.

Cross generation related changes (comparing group 17 vs 11, 18 vs 12, 19 vs 13, 20 vs 14, 21 vs 15, 22 vs 16): No clear cross generation related changes were seen in any evaluated clinical pathology parameter.

1.3.3 Liver F2 – Pathology – microscopic observations

Administration of carbon tetrachloride in animals from groups 12, 15, 18 and 21, dosed at 8% CCl_4 resulted in mild liver changes (degenerative and fibrotic), affecting in similar manner all animals, regardless of the generation trait. Consistently, administration of higher doses of carbon tetrachloride in animals from groups 13, 16, 19, and 22, dosed at 50% CCl_4, resulted in a combination of severe liver changes (degenerative, fibrotic and hyperplastic) affecting in a similar manner all animals, with no generational trait modulation.

Liver findings were described as centrilobular fibrosis, hepatocyte vacuolation, hepatocellular degeneration/necrosis, hepatocellular regenerative hyperplasia, hepatocyte karyomegaly, hepatocyte altered cellular foci, biliary hyperplasia and pigment deposits. In details:

1) Minimal to marked centrilobular fibrosis, often bridging and dissecting lobules, was seen in all animals receiving CCl_4, dose-related in terms of severity, however with similar severity within the same dose level, regardless of the generational traits. In animals receiving 8% CCl_4, the finding appeared to run along the "pericentral areas" of the hepatic acinus, rather than centrilobular space of the hepatic lobule. In animals receiving 50% CCl_4, the fibrosis was rather diffuse, bridging centrilobular to periportal spaces and dissecting the lobules without a clear pattern.

Table 1-3 Incidence and severity of CCl_4-liver changes – Fibrosis

**Organ** Sex	Males											
Dose (oil:CCl_4)	100:0	92:8	50:50	100:0	92:8	50:50	100:0	92:8	50:50	100:0	92:8	50:50
Group no	11	12	13	14	15	16	17	18	19	20	21	22
**Liver**	(10)	(10)	(10)	(10)	(10)	(10)	(9)	(10)	(9)	(10)	(9)	(10)
Fibrosis, centrilobular	**0**	**10**	**10**	**0**	**10**	**10**	**0**	**10**	**9**	**0**	**9**	**10**
Mild	-	6	2	-	6	1	-	7	-	-	6	2
Moderate	-	4	4	-	4	6	-	3	6	-	3	3
Marked	-	-	4	-	-	3	-	-	3	-	-	5

2) Minimal to marked hepatocyte vacuolation, macro-microvesicular and mainly present in centrilobular (pericentral) position, was seen in all CCl_4-treated animals. Minimal to mild microvesicular vacuolation, affecting hepatocytes in periportal position, was rather seen in some control animals, and control animals were also less affected in terms of incidence and severity.

Table 1-4 Incidence and severity of CCl_4-liver changes – Vacuolation

**Organ** Sex	Males											
Dose (oil:CCl_4)	100:0	92:8	50:50	100:0	92:8	50:50	100:0	92:8	50:50	100:0	92:8	50:50
Group no	11	12	13	14	15	16	17	18	19	20	21	22
**Liver**	(10)	(10)	(10)	(10)	(10)	(10)	(9)	(10)	(9)	(10)	(9)	(10)
Microvacuolation, hepatocyte	**3**	**0**	**0**	**2**	**0**	**0**	**2**	**0**	**0**	**5**	**0**	**0**
Minimal	1	-	-	2	-	-	2	-	-	4	-	-
Mild	2	-	-	-	-	-	-	-	-	1	-	-
Vacuolation, hepatocyte	**0**	**10**	**10**	**0**	**10**	**10**	**0**	**10**	**9**	**0**	**9**	**10**
Minimal	-	7	-	-	5	1	-	7	1	-	8	-
Mild	-	3	6	-	5	4	-	3	4	-	1	8
Moderate	-	-	3	-	-	5	-	-	3	-	-	2
Marked	-	-	1	-	-	-	-	-	1	-	-	-

3) Minimal to severe hepatocyte degeneration/necrosis, vacuolar, with hepatocyte displaying swelling and ballooning degeneration, mainly in centrilobular position, up to hepatocellular necrosis and loss, was seen in all CCl_4-treated animals, and appeared dose-related in terms of severity. In animals receiving 8% CCl_4, no differences were noted in similar manner for all animals, regardless of the generation trait. In animals receiving 50% CCl_4, group 13 appeared to be slightly more affected than the remaining CCl_4-treated groups, in terms of severity.

Table 1-5 Incidence and severity of CCl_4-liver changes – Degeneration/necrosis

**Organ** Sex	Males											
Dose (oil:CCl_4)	100:0	92:8	50:50	100:0	92:8	50:50	100:0	92:8	50:50	100:0	92:8	50:50
Group no	11	12	13	14	15	16	17	18	19	20	21	22
**Liver**	(10)	(10)	(10)	(10)	(10)	(10)	(9)	(10)	(9)	(10)	(9)	(10)
Degeneration/ necrosis, hepatocyte	**0**	**10**	**10**	**0**	**10**	**10**	**0**	**10**	**9**	**0**	**9**	**10**
Minimal	-	4	-	-	5	-	-	6	-	-	5	1
Mild	-	4	2	-	4	4	-	3	5	-	3	1
Moderate	-	2	4	-	1	3	-	1	3	-	1	5
Marked	-	-	-	-	-	2	-	-	1	-	-	2
Severe	-	-	4	-	-	1	-	-	-	-	-	1

4) Minimal to severe biliary hyperplasia, with biliary channels running inside the fibrotic septa was seen only in rats receiving 50% CCl_4. Rats from group 13 appeared to be slightly less affected than the remaining CCl_4-treated groups, in terms of severity. One control animal from group 11 and one animal from group 21 had a minimal focal biliary hyperplasia, which was clearly different from the finding in the treated group, as it was focal in nature and not associated with fibrosis.

Table 1-6 Incidence and severity of CCl_4-liver changes – Biliary hyperplasia

**Organ** Sex	Males											
Dose (oil:CCl_4)	100:0	92:8	50:50	100:0	92:8	50:50	100:0	92:8	50:50	100:0	92:8	50:50
Group no	11	12	13	14	15	16	17	18	19	20	21	22
**Liver**	(10)	(10)	(10)	(10)	(10)	(10)	(9)	(10)	(9)	(10)	(9)	(10)
Hyperplasia, biliary	**1**	**0**	**10**	**0**	**0**	**10**	**0**	**0**	**9**	**0**	**1**	**10**
Minimal	1	-	-	-	-	-	-	-	-	-	1	1
Mild	-	-	4	-	-	2	-	-	2	-	-	3
Moderate	-	-	5	-	-	4	-	-	4	-	-	1
Marked	-	-	1	-	-	1	-	-	1	-	-	1
Severe	-	-	-	-	-	3	-	-	2	-	-	4

5) Minimal to moderate hepatocellular regenerative hyperplasia, was seen in the majority of the rats receiving 50% CCl_4. Additional changes, consistent with a hyperplastic process were also seen in most CCl_4 treated animals, and consisted of minimal to moderate hepatocyte karyomegaly, scattered through the parenchyma, and minimal to moderate hepatocyte altered cell foci. No obvious difference among the different generational traits was seen. None of these findings were seen in animals receiving 8% CCl_4.

Table 1-7 Incidence and severity of CCl_4-liver changes – hyperplasia

**Organ** Sex	Males											
Dose (oil:CCl_4)	100:0	92:8	50:50	100:0	92:8	50:50	100:0	92:8	50:50	100:0	92:8	50:50
Group no	11	12	13	14	15	16	17	18	19	20	21	22
**Liver**	(10)	(10)	(10)	(10)	(10)	(10)	(9)	(10)	(9)	(10)	(9)	(10)
Hyperplasia, hepatocellular, regenerative	**0**	**0**	**10**	**0**	**0**	**10**	**0**	**0**	**9**	**0**	**0**	**10**
Minimal	-	-	1	-	-	-	-	-	-	-	-	1
Mild	-	-	8	-	-	9	-	-	9	-	-	5
Moderate	-	-	1	-	-	1	-	-	-	-	-	4
Karyomegaly, hepatocyte	**0**	**0**	**10**	**0**	**0**	**10**	**0**	**0**	**9**	**0**	**0**	**10**
Minimal	-	-	2	-	-	2	-	-	-	-	-	1
Mild	-	-	8	-	-	5	-	-	8	-	-	7
Moderate	-	-	-	-	-	3	-	-	1	-	-	2
Altered cell foci, hepatocyte	**0**	**0**	**10**	**0**	**0**	**10**	**0**	**0**	**9**	**0**	**0**	**10**
Minimal	-	-	9	-	-	8	-	-	8	-	-	5
Mild	-	-	-	-	-	2	-	-	1	-	-	3
Moderate	-	-	1	-	-	-	-	-	-	-	-	2

6) Minimal to mild histiocytic pigments, usually interstitially placed within fibrous septa, was seen in the majority of the rats receiving CCl_4, at both doses, with a dose-relationship in terms of incidence and severity. No obvious difference among the different generational traits was seen, with group 21 showing the lowest incidence.

Table 1-8 Incidence and severity of CCl_4-liver changes – pigment

**Organ** Sex	Males											
Dose (oil:CCl_4)	100:0	92:8	50:50	100:0	92:8	50:50	100:0	92:8	50:50	100:0	92:8	50:50
Group no	11	12	13	14	15	16	17	18	19	20	21	22
**Liver**	(10)	(10)	(10)	(10)	(10)	(10)	(9)	(10)	(9)	(10)	(9)	(10)
Pigment deposit, histiocytic	**0**	**6**	**10**	**0**	**6**	**10**	**0**	**5**	**9**	**0**	**3**	**10**
Minimal	-	6	6	-	6	5	-	5	2	-	2	2
Mild	-	-	4	-	-	5	-	-	7	-	1	8
