# Supplementary material for: Unaltered hepatic wound healing response in male rats with ancestral liver injury
Source: Nat Commun. 2023 Oct 10;14:6353. doi: 10.1038/s41467-023-41998-w (PMC10564731; doi:10.1038/s41467-023-41998-w)
Supplement: Supplementary file 7 — Reporting Summary [file 41467_2023_41998_MOESM7_ESM.pdf]

nature portfolio

Corresponding author(s): Remi Terranova

Last updated by author(s): Sep 15, 2023

Reporting Summary

Nature Portfolio wishes to improve the reproducibility of the work that we publish. This form provides structure for consistency and transparency in reporting. For further information on Nature Portfolio policies, see our Editorial Policies and the Editorial Policy Checklist.

Statistics

For all statistical analyses, confirm that the following items are present in the figure legend, table legend, main text, or Methods section.

n/a	Confirmed	
☐	☒	The exact sample size (*n*) for each experimental group/condition, given as a discrete number and unit of measurement
☐	☒	A statement on whether measurements were taken from distinct samples or whether the same sample was measured repeatedly
☐	☒	The statistical test(s) used AND whether they are one- or two-sided<br>*Only common tests should be described solely by name; describe more complex techniques in the Methods section.*
☐	☒	A description of all covariates tested
☐	☒	A description of any assumptions or corrections, such as tests of normality and adjustment for multiple comparisons
☐	☒	A full description of the statistical parameters including central tendency (e.g. means) or other basic estimates (e.g. regression coefficient) AND variation (e.g. standard deviation) or associated estimates of uncertainty (e.g. confidence intervals)
☐	☒	For null hypothesis testing, the test statistic (e.g. *F*, *t*, *r*) with confidence intervals, effect sizes, degrees of freedom and *P* value noted<br>*Give P values as exact values whenever suitable.*
☒	☐	For Bayesian analysis, information on the choice of priors and Markov chain Monte Carlo settings
☒	☐	For hierarchical and complex designs, identification of the appropriate level for tests and full reporting of outcomes
☐	☒	Estimates of effect sizes (e.g. Cohen's *d*, Pearson's *r*), indicating how they were calculated

Our web collection on statistics for biologists contains articles on many of the points above.

Software and code

Policy information about availability of computer code

Data collection	All data has been produced and analyzed in-house.<br>Rat liver single cell RNAseq data was imported from GSE137869 (https://www.ncbi.nlm.nih.gov/geo/query/acc.cgi?acc=GSE137869) as detailed in the Methods of the manuscript and the data section below.
Data analysis	All visualizations and analyses were performed in R (version 4.1.1). Custom analysis scripts generated within the course of this work are publicly available and deposited under: https://github.com/jperner/TGmanuscript.

For manuscripts utilizing custom algorithms or software that are central to the research but not yet described in published literature, software must be made available to editors and reviewers. We strongly encourage code deposition in a community repository (e.g. GitHub). See the Nature Portfolio guidelines for submitting code & software for further information.

Data

Policy information about availability of data

All manuscripts must include a data availability statement. This statement should provide the following information, where applicable:

- Accession codes, unique identifiers, or web links for publicly available datasets
- A description of any restrictions on data availability
- For clinical datasets or third party data, please ensure that the statement adheres to our policy

RNA sequencing data originating from this study have been deposited in NCBI GEO under the accession code: GSE229524 (ADD LINK GEO) for liver and kidney

samples. Single cell RNA sequencing data analyzed in this study was accessed from NCBI GEO under the accession code: GSE137869 (https://www.ncbi.nlm.nih.gov/geo/query/acc.cgi?acc=GSE137869) and the sample sets GSM4331834 (young male) and GSM4331835 (old male) were selected and processed from count matrix as stated in the Methods section. A Data availability statement has been added to the manuscript and Source data are provided with this paper (Source Data Supplementary File).

Human research participants

Policy information about studies involving human research participants and Sex and Gender in Research.

Reporting on sex and gender	No human research participants were part of this study.
Population characteristics	No human research participants were part of this study.
Recruitment	No human research participants were part of this study.
Ethics oversight	No human research participants were part of this study.

Note that full information on the approval of the study protocol must also be provided in the manuscript.

Field-specific reporting

Please select the one below that is the best fit for your research. If you are not sure, read the appropriate sections before making your selection.

- [x] Life sciences
- [ ] Behavioural & social sciences
- [ ] Ecological, evolutionary & environmental sciences

For a reference copy of the document with all sections, see nature.com/documents/nr-reporting-summary-flat.pdf

Life sciences study design

All studies must disclose on these points even when the disclosure is negative.

Sample size	Sample size calculations were not performed for experiments in this study.<br>10 animals per ancestral cohort (n=4) and dose-group (n=3) were evaluated for F2 phenotypic characterization (total of 120 F2 animals).
Data exclusions	One animal of the F0 generation (animal 2005) and two animals in the F2 generation (Vehicle control treated animal 17005 and CCl4 treated animal 19003) did not tolerate the treatment and were excluded from the analyses. Animal 20003 was excluded from IHC analysis for technical reasons.
Replication	This study aims at reproducing previously published work using similar in vivo multi-generation paradigm. We carefully considered important study features such as animal house and care, high study power, cross-generational pedigree tracing and representation and careful staggering and randomization schemes for F2 dose-response treatment, collection and evaluation. Detailed description of study design and all study features can be found in the manuscript for independent replication.
Randomization	Randomization was applied at various stages of the study, in vivo and throughout various molecular evaluations. Briefly, in vivo pedigree tracing was applied through the F0-F2 generations. Given the large number of F2 animals (4 ancestral cohorts and 3 treatment groups, n=120 in total) and to avoid treatment or collection biases, we used a carefully designed staggering and randomization scheme for F2 dose-response treatment, necropsy, collection and evaluation, including at molecular levels. Detailed, animal-level information is provided in Supplementary Methods.
Blinding	Investigators were not blinded to group allocation during data collection and analysis. Knowledge of the F0-F1 liver fibrosis history represents the core anchor to interpreting pathology and molecular effects and evaluating the transgenerational phenomenon.

Reporting for specific materials, systems and methods

We require information from authors about some types of materials, experimental systems and methods used in many studies. Here, indicate whether each material, system or method listed is relevant to your study. If you are not sure if a list item applies to your research, read the appropriate section before selecting a response.

Materials & experimental systems

n/a	Involved in the study	
☐	☒	Antibodies
☒	☐	Eukaryotic cell lines
☒	☐	Palaeontology and archaeology
☐	☒	Animals and other organisms
☒	☐	Clinical data
☒	☐	Dual use research of concern

Methods

n/a	Involved in the study	
☒	☐	ChIP-seq
☒	☐	Flow cytometry
☒	☐	MRI-based neuroimaging

Antibodies

Antibodies used	- Mouse monoclonal anti-alpha Smooth-muscle actin (aSMA), clone 1A4, provider: DAKO, reference: M0851, batch 41327852<br>- DISCOVERY OmniMap anti-Mouse HRP, provider: Roche Diagnostics, reference: 05269652001, catalog number: 760-4310
Validation	The specificity of the alpha-SMA antibody was tested by the provider using SDS-PAGE immunoblotting of an alpha-smooth muscle actin (SMA). The antibody was able to detect the band corresponding to alpha-SMA. For usage on rat tissue in this study, a Blast sequence alignment was performed and 100% sequence homology was found. The staining results on rat liver are consistent with smooth muscle cells staining. The secondary antibody is certified and commercialized by Ventana.

Animals and other research organisms

Policy information about studies involving animals; ARRIVE guidelines recommended for reporting animal research, and Sex and Gender in Research

Laboratory animals	Animals used in this study were outbred Sprague Dawley rats. Ordered F0 (males, females) and F1 (females) were supplied by Charles River Laboratories Raleigh, NC approximately 10 weeks of age (Supplementary Methods, Table 1-4). Throughout the study males were treated at 9-11 weeks of age. Detailed study schedules for all three generations are provided in Supplementary Methods (F0: Table 1-8, F1: Table 1-9, F2: Table 1-10).
Wild animals	The study did not involve wild animals.
Reporting on sex	This multi-generation study exclusively evaluates male transmission effects (as originally reported). Naive females were used for breeding F1 and F2 animals but the transgenerational phenomenon not evaluated in females.
Field-collected samples	The study did not involve samples collected from the field.
Ethics oversight	The study has been conducted in accordance with the Novartis Animal Care and Use Committee. All procedures in this study are in compliance with the Animal Welfare Act, the Guide for the Care and Use of Laboratory Animals, and the Office of Laboratory Animal Welfare.

Note that full information on the approval of the study protocol must also be provided in the manuscript.
